# Changes in Kidney and Liver Volumes in Patients With Autosomal Dominant Polycystic Kidney Disease Before and After Dialysis Initiation

**DOI:** 10.1016/j.mayocpiqo.2022.12.005

**Published:** 2023-01-20

**Authors:** Tatsuya Suwabe, Yoshifumi Ubara, Yuki Oba, Hiroki Mizuno, Daisuke Ikuma, Masayuki Yamanouchi, Akinari Sekine, Kiho Tanaka, Eiko Hasegawa, Junichi Hoshino, Naoki Sawa

**Affiliations:** a Department of Nephrology, Toranomon Hospital, Tokyo and Kawasaki, Japan; b Okinaka Memorial Institute for Medical Research, Toranomon Hospital, Tokyo, Japan; c Department of Nephrology, Tokyo Women’s Medical University, Tokyo, Japan

**Keywords:** ACDK, acquired cystic disease of the kidney, ADPKD, autosomal dominant polycystic kidney disease, ALB, albumin, BMI, body mass index, BP, blood pressure, CI, confidence interval, CT, computed tomography, eGFR, estimated glomerular filtration rate, ESKD, end-stage kidney disease, HD, hemodialysis, HR, heart rate, MRI, magnetic resonance imaging, PD, peritoneal dialysis, PKD, polycystic kidney disease, PLD, polycystic liver disease, TAE, transcatheter arterial embolization, TKV, total kidney volume, TLV, total liver volume, UN, urea nitrogen

## Abstract

**Objective:**

To examine the changes in total kidney volume (TKV) and total liver volume (TLV) before and after dialysis initiation in patients with autosomal dominant polycystic kidney disease.

**Patients and Methods:**

This was a retrospective, single-center cohort study to investigate the changes in TKV and TLV before and after dialysis initiation, along with influencing factors, using linear mixed models. We enrolled 95 patients with autosomal dominant polycystic kidney disease (85 receiving hemodialysis [HD] and 10 receiving peritoneal dialysis [PD]) who began receiving dialysis at Toranomon Hospital from January 1, 2008, to December 31, 2020.

**Results:**

The least squares mean TKV ratio (TKV at each time point/TKV at dialysis initiation) was 63.8% (95% confidence interval [CI], 54.7%-72.9%) at 6 years before dialysis initiation and 95.5% (95% CI, 82.9%-108.2%) at 6 years after dialysis initiation (*P*<.001). A multivariate linear mixed model analysis revealed that dialysis style (HD or PD) had the strongest effect on changes in TKV (*P*=.002). The least squares mean TLV ratio was 98.2% (95% CI, 88.4%-108.0%) at 6 years before dialysis initiation and 95.7% (95% CI, 85.2%-106.2%) at 6 years after dialysis initiation (*P*=.01). Although PD did not have significant effects on changes in TLV (*P*=.27), the changes in TLV were greater in patients on PD than in those on HD.

**Conclusion:**

The TKV increased until dialysis initiation and generally decreased after dialysis initiation. The TLV continued to increase even after dialysis initiation, however, changes in the TLV significantly decreased after dialysis initiation. The increases in TKV and TLV were greater in patients on PD than in those on HD.

Autosomal dominant polycystic kidney disease (ADPKD) is a common hereditary disorder and the fourth leading cause of end-stage kidney disease (ESKD) in adults.^1^, ^2^, ^3^ ADPKD is characterized by progressive development and growth of cysts that cause kidney enlargement and distortion, kidney function impairment, and—in many patients—ESKD. The speed of the progression of polycystic kidney disease (PKD) varies among patients; however, the kidney volume often gradually increases with decreasing renal function. Kidney volume is reportedly the most robust marker for the prediction of ADPKD progression.^4^ Increases in kidney volume exhibit growing associations with decreasing renal function until the onset of ESKD in patients with ADPKD. To our knowledge, there have been few reports regarding kidney volume after dialysis initiation. A few studies have reported conflicting findings concerning changes in kidney volume among patients with ADPKD receiving dialysis.^5^, ^6^, ^7^ One group reported that most patients with ADPKD receiving hemodialysis (HD) found an initial rapid reduction in the total kidney volume (TKV), followed by a slow increase.^5^ However, another group reported that the TKV increased after HD initiation in some patients.^8^ Importantly, there are fewer reports regarding the total liver volume (TLV) after dialysis initiation. Thus, TKV and TLV have not been fully characterized in patients with ADPKD and ESKD. Here, we investigated the changes in TKV and TLV before and after dialysis initiation in patients with ADPKD and then examined the potential influencing factors for both TKV and TLV.

## Methods

### Study Design, Population and Setting

This was a retrospective, single-center cohort study. This study was reviewed and approved by the ethics committee of Toranomon Hospital in June 2020. This study was limited to a retrospective review of imaging tests and medical records for a subset of enrolled patients. Therefore, specific informed consent was not required for this study. We also used an opt-out approach that involved posting the study information on our hospital’s website. All patients who began receiving dialysis because of ESKD caused by ADPKD in Toranomon Hospital (Tokyo and Kajigaya) from January 2008 to December 2020 were enrolled in this study. This study period was defined because electronic images were available since November 2007 at our hospital. These patients were identified from the database of the Department of Nephrology, Toranomon Hospital, which was updated by a research assistant each time a patient began dialysis. All patients were adults (aged ≥20 years) who met the criteria for the diagnosis of ADPKD defined by Pei et al^9^ and the Progressive Renal Disease Research unit from the Ministry of Health, Labour and Welfare of Japan (Supplemental Table 1, available online at http://www.mcpiqojournal.org). We excluded patients who did not undergo abdominal computed tomography (CT) or magnetic resonance imaging (MRI) during the year of dialysis initiation and at least once more than 1 year before and after dialysis initiation. Patients were censored at the time point at which they underwent transcatheter arterial embolization (TAE) or cyst drainage for polycystic kidneys or livers because such procedures may affect TKV or TLV. Patients were also censored at the time point at which they underwent kidney transplantation. Patients on peritoneal dialysis (PD) were censored at the time point at which they were changed to HD or when they began weekly HD combined with PD.

### Clinical and Laboratory Assessments

All patients who began receiving dialysis at our hospital were required to be hospitalized; this allowed careful observation after dialysis initiation and adjustment of dialysis conditions. Clinical features were recorded at dialysis initiation. Body weight and blood pressure (BP) were also recorded daily, including at the beginning of each HD term. Dry weight was used as the body weight of patients on HD. The BP was measured using an automatic device with the patient in the sitting position; the mean of 3 BP readings measured in the morning on a nondialysis day was used for analysis. The body mass index (BMI) was calculated as the body weight in kilograms divided by the square of the height in meters. Blood tests were performed at the beginning of HD in patients on HD. All laboratory tests were conducted using automated, standardized methods at our hospital within 24 hours after collection of blood samples. Leg edema was identified by each attending physician. The estimated glomerular filtration rate (eGFR) was calculated in accordance with the Japanese coefficient of the modified isotope dilution mass spectrometry Modification of Diet in Renal Disease study.^10^

### Imaging Studies

Abdominal CT or MRI was performed using a previously reported method.^11^ The kidney and liver volumes were determined using the Vincente software, version 4 (Fujifilm Co., Japan), by a single group of medical staff members. The patients usually underwent abdominal CT or MRI immediately before dialysis initiation; however, the frequencies of these imaging tests varied among the patients. We reviewed all imaging test results and measured the TKV or TLV in each patient from beginning in 2008. Imaging tests within 6 months of each time point were eligible analysis at each time point. If the patients underwent renal-TAE, we did not review subsequent TKV findings. If the patients underwent hepatic-TAE, we did not review subsequent TLV findings. We also did not review images after cyst drainage, kidney transplantation, or nephrectomy. No patients underwent liver transplantation.

The TKV or TLV ratio at each time point was calculated as follows: TKV (or TLV) ratio=TKV (or TLV) at each time point/TKV (or TLV) at dialysis initiation. The TKV (or TLV) ratio at each time point was defined as the ratio of the value at the time point to the TKV (or TLV) value at dialysis initiation. A ratio of 100% indicated TKV (or TLV) values equal to those at dialysis initiation, a ratio of less than 100% indicated a smaller TKV (or TLV) value than that at dialysis initiation, and a ratio of more than 100% indicated a larger TKV (or TLV) value than that at dialysis.

We classified all enrolled patients by their height-adjusted TKV (HtTKV) (in accordance with the Mayo classification system).^4^ We also classified all enrolled patients by their height-adjusted total liver volume (HtTLV) quartiles.

### Statistical Analyses

To estimate the mean values of TKV ratio, TLV ratio, and systolic BP at each time point, least squares mean values and 95% confidence intervals (CIs) were calculated using a linear mixed model. The least squares mean TKV (or TLV) ratio indicated the estimated marginal means of the TKV (or TLV) ratio at each time point; these were the group means after having controlled for a covariate. In this model, time (a nominal measure) was set as both a fixed and repeated effect, whereas patient identification number was set as a random effect. Additionally, our covariance model used compound symmetry. Furthermore, to estimate the changes in the slope coefficients of TKV ratio, TLV ratio, and systolic BP curves before and after dialysis imitation, a linear mixed model analysis was performed, considering the interaction between the timing of dialysis initiation and time (a continuous measure).

Linear mixed model analyses with interaction between dialysis style (HD or PD) and time were used to estimate the difference in the slope coefficients of TKV ratio, TLV ratio, and systolic BP curves according to dialysis style. Again, the patients were used as a random factor in this model, and composite symmetry was used.

Furthermore, the effects of each predictor variable on the slope coefficients of the TKV and TLV ratios were examined in a linear mixed model. Predictive variables that were significant in a univariate analysis were selected for the multivariate analysis using linear mixed models. In these models, TKV or TLV was a response variable; time (a continuous measure), predictive variables (eg, age, sex, BMI, systolic BP, diastolic BP, heart rate (HR), hemoglobin, serum total protein, serum albumin (Alb), serum C-reactive protein, eGFR, serum urea nitrogen (UN), dialysis style [HD vs PD], HtTKV, and HtTLV), and interaction predictive variables and time were fixed effects; and the patients were random effects.

Normally distributed baseline variables were summarized as means ± SDs, whereas nonnormally distributed numeric baseline variables were summarized as medians and interquartile ranges. For quantitative variables, differences between 2 groups were assessed using the Student t-test or Mann-Whitney U test. For categorical variables, differences between groups were assessed using the χ^2^ test or Fisher exact test for discrete variables.

All statistical analyses were performed using SPSS, version 22.0, for Windows (IBM Japan). All *P* values were 2 tailed; *P* values less than .05 were considered statistically significant.

## Results

**Figure 1:**
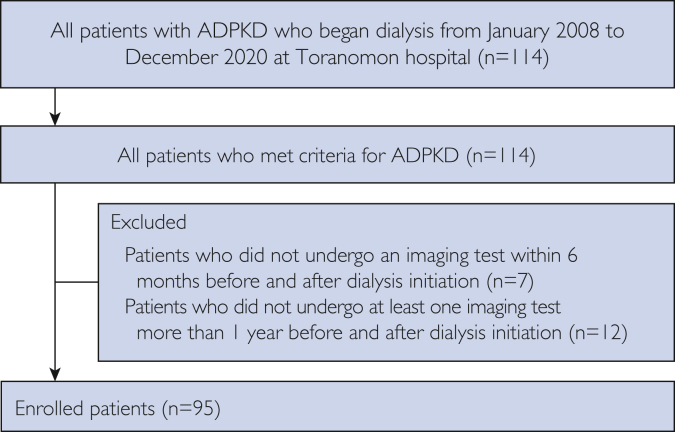
Flowchart showing enrollment of all patients who began receiving dialysis from January 2008 to December 2018 at Toranomon Hospital.

In total, 114 patients with ADPKD began receiving dialysis at our hospital from January 2008 to December 2020. Among them, 7 patients who did not undergo an imaging test during the year of dialysis initiation and 12 patients who did not undergo abdominal imaging tests at 2 or more time points were excluded (Figure 1). Overall, 95 patients (28 men and 67 women), with a mean age of 58.9±10.3 years, were enrolled (Table 1). Eighty-five patients received HD, and 10 patients received PD. No patients had undergone kidney transplantation before dialysis initiation. Of the 10 patients who were on PD, 2 had begun weekly HD combined with PD; they were censored at the time point at which they began weekly HD combined with PD. The distributions of height-adjusted TKV, HtTLV, and age at dialysis initiation are presented in Supplemental Figure 1A and B (available online at http://www.mcpiqojournal.org).

**Table 1:** Clinical Characteristics of Enrolled Patients^a^

	All patients	Patients on HD	Patients on PD	*P* value^b^
Number (M/F)	95 (28/67)	85 (23/62)	10 (5/5)	.15
Age (y), mean ± SD	58.9±10.3	59.8±10.1	51.1±8.6	.02
BMI (kg/m^2^), mean ± SD	23.2±3.0	23.1±3.3	21.7±2.3	.24
Total kidney volume (mL), median (IQR)	3038.2 (2092.1-4489.8)	3013.1 (2152.1-4154.4)	4031.3 (1898.3-5097.9)	.73
Height-adjusted total kidney volume (mL/m), median (IQR)	1868.4 (1367.8-2685.5)	1863.5 (1376.0-2463.1)	2451.5 (1236.7-2947.2)	.81
Distribution of Mayo classification	1A; 2 (2.1%) 1B; 9 (9.5%) 1C; 40 (42.1%) 1D; 27 (28.4%) 1E; 17 (17.9%)	1A; 2 (2.4%) 1B; 9 (10.6%) 1C; 35 (41.2%) 1D; 27 (31.8%) 1E; 12 (14.1%)	1A; 0 (0.0%) 1B; 0 (0.0%) 1C; 5 (50.0%) 1D; 0 (0.0%) 1E; 5 (50.0%)	.01
Liver volume (mL), median (IQR)	2604.6 (1634.4-4000.0)	2959.7 (1706.9-4440.4)	1665.6 (1565.6-1879.2)	.02
Height-adjusted total liver volume (mL/m), median (IQR)	1599.4 (1053.2-2683.7)	1872.6 (1075.6-3088.7)	1062.5 (935.0-1118.7)	.01
Systolic blood pressure (mm Hg), mean ± SD	139.0±20.0	140.0±20.1	130.3±13.2	.14
Diastolic blood pressure (mm Hg), mean ± SD	82.2±11.4	82.2±11.6	82.4±11.0	.95
Heart rate (/min)	78.3±13.6	78.0±13.9	81.0±11.1	.51
Blood test				
Hb (g/μL), mean ± SD	9.8±1.9	9.7±1.9	11.3±1.9	.01
TP (g/dL), mean ± SD	6.6±0.6	6.6±0.5	6.6±0.7	.64
Alb (g/dL), mean ± SD	3.0±0.5	3.0±0.5	3.3±0.5	.10
Cr (mg/dL), mean ± SD	7.5±2.8	7.3±2.7	8.8±3.7	.12
UN (mg/dL), mean ± SD	75.9±24.9	75.8±24.8	76.8±27.5	.91
CRP (mg/L), median (IQR)	0.085 (0.022-0.251)	0.093 (0.021-0.260)	0.073 (0.020-0.228)	.71

a Alb, albumin; BMI, body mass index; Cr, creatinine; CRP, C-reactive protein; F, female; Hb, hemoglobin; HD, hemodialysis; IQR, interquartile range; M, male; PD, peritoneal dialysis; TP, total protein; UN, urea nitrogen.

b Comparison of patients on HD and those on PD.

**Figure 2:**
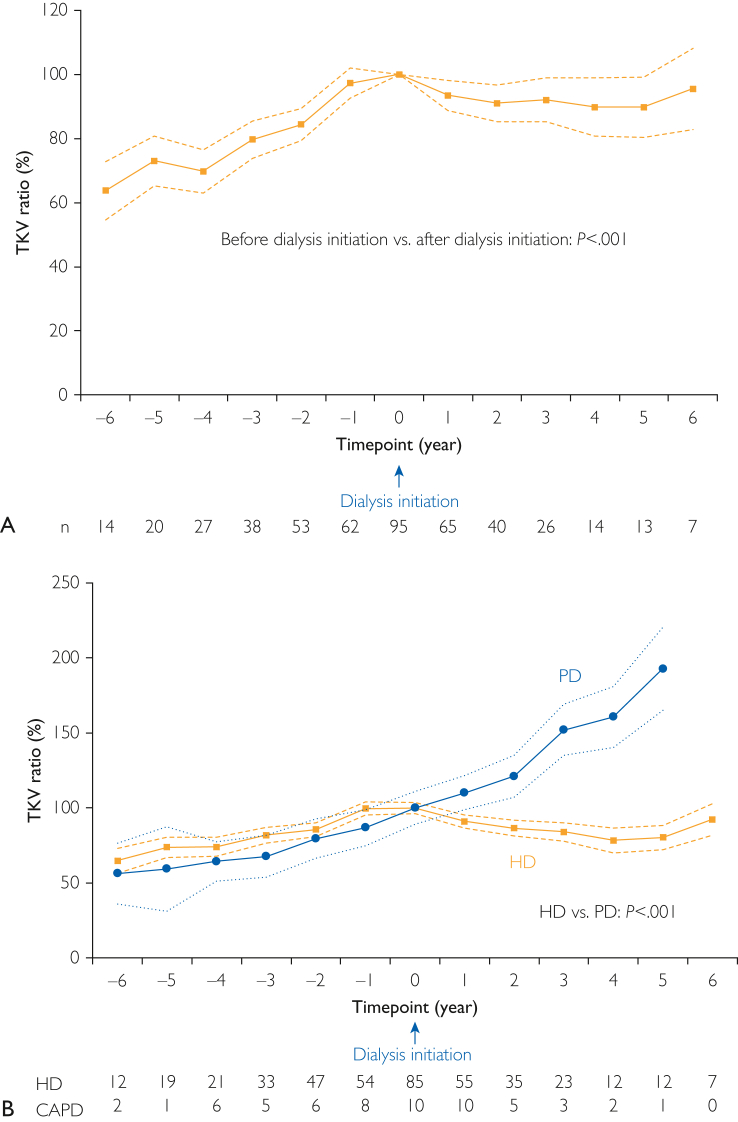
(A) Least squares mean (95% confidence interval) of the total kidney volume ratio at each time point using a linear mixed model to report significant changes after dialysis initiation. ∗*P*<.001 comparing the slope of total kidney volume ratio between before and after dialysis initiation. (B) Linear mixed model analysis reported that the least squares mean (95% confidence interval) of the total kidney volume ratio at each time point was significantly lower in patients on HD than in patients on PD (*P*<.001). HD, hemodialysis; PD, peritoneal dialysis; TKV, total kidney volume.

The changes in the TKV ratio at each time point are shown in Figure 2A. At 6 years before dialysis initiation, the least squares mean TKV ratio was 63.8% (95% CI, 54.7%-72.9%). At 6 years after dialysis initiation, the least squares mean TKV ratio was 95.5% (95% CI, 82.9%-108.2%). The changes in the TKV ratio were significantly lower after dialysis initiation than those before dialysis initiation (*P*<.001) (Supplemental Table 2, available online at http://www.mcpiqojournal.org). The actual measured TKV of each patient on HD and PD at each time point is presented in Supplemental Figure 2A and B (available online at http://www.mcpiqojournal.org).

The univariate linear mixed model analysis revealed that age, sex, serum Alb, Mayo classification, HtTLV, TLV quartile category, and PD had significant effects on the changes in TKV (Table 2). The multivariate linear mixed model analysis—using variables that were significant in the univariate analysis—revealed that sex and PD had significant effects on the changes in the TKV ratio (P=.02, .002, respectively). The TKV ratios at all time points, stratified according to HD and PD, are shown in Figure 2B. The TKV ratio after dialysis initiation was significantly higher in the patients on PD than in those on HD (*P*<.001) (Supplemental Table 3, available online at http://www.mcpiqojournal.org). The TKV ratio in the patients on PD continued to increase even after dialysis initiation. The TKV ratios at all time points, stratified according to male and female sex, are shown in Supplemental Figure 2C. The TKV ratio after dialysis initiation was significantly higher in men than in women (*P*<.001). The changes in the TKV ratio according to the Mayo classification group are presented in Supplemental Figure 2D. There were significant differences in the changes in the TKV ratio before and after dialysis initiation among the 4 groups (*P*<.001). The TKV in patients who were classified as Mayo class 1E increased rapidly before and after dialysis initiation.

**Table 2:** The Changes (95% Confidence Intervals) in Slope Coefficients of Total Kidney Volume Curves by Predictive Variables in Univariate and Multivariable Linear Mixed Model Analyses^a^

	Change in TKV (%/y)								
	Univariate analysis				Multivariate analysis^b^				
	Regression coefficient	SE	Beta	*P* value	Regression coefficient	SE	Beta	*P* value	VIF
Clinical characteristics at dialysis initiation									
Age	−0.40	0.14	−0.325	.004	−0.14	0.20	−0.110	.50	2.439
Male (vs female)	12.02	2.970	0.426	<.001	8.55	3.56	0.294	.02	1.422
BMI	−0.21	0.53	−0.045	.70					
Systolic BP	−0.09	0.08	−0.133	.26					
Diastolic BP	0.08	0.14	0.072	.54					
HR	−0.05	0.12	−0.054	.65					
Hb	1.07	0.76	0.166	.16					
Serum TP	2.11	2.78	0.090	.45					
Serum Alb	8.29	3.27	0.288	.01	3.11	4.06	0.088	.45	1.249
Serum CRP	−0.54	0.40	−0.161	.18					
eGFR	−0.58	0.60	−0.114	.33					
Serum UN	0.03	0.07	0.060	.62					
HtTKV (per 100 mL)	0.18	0.12	0.178	.13					
Mayo classification (per 1 grade)	3.47	1.47	0.265	.02	−0.93	2.40	−0.063	.70	2.487
HtTLV (per 100 mL)	−0.30	0.10	−0.391	.003	n.e.				
HtTLV (per 1 category)^c^	−5.38	1.49	−0.445	.001	−2.75	1.51	−0.218	.08	1.375
PD (vs HD)	20.20	3.96	0.510	<.001	15.58	4.70	0.406	.002	1.426

a Alb, albumin; BMI, body mass index; BP, blood pressure; CRP, C-reactive protein; eGFR, estimated glomerular filtration rate; Hb, hemoglobin; HD, hemodialysis; HR, heart rate; HtTKV, height-adjusted total kidney volume; HtTLV, height-adjusted total liver volume; n.e.: not entered because of collinearity; PD, peritoneal dialysis; SE, standard error; TP, total protein; TKV, total kidney volume; UN, urea nitrogen; VIF, variance inflation factor.

b The significant variables in the univariate analysis were selected for the multivariate analysis.

c Patients were grouped by HtTLV as follows: quartile 1, <1053.2 mL/m; quartile 2, 1053.2 to <1599.3 mL/m; quartile 3, 1599.3 to <2683.6 mL/m; and quartile 4, ≥2683.6 mL/m.

**Figure 3:**
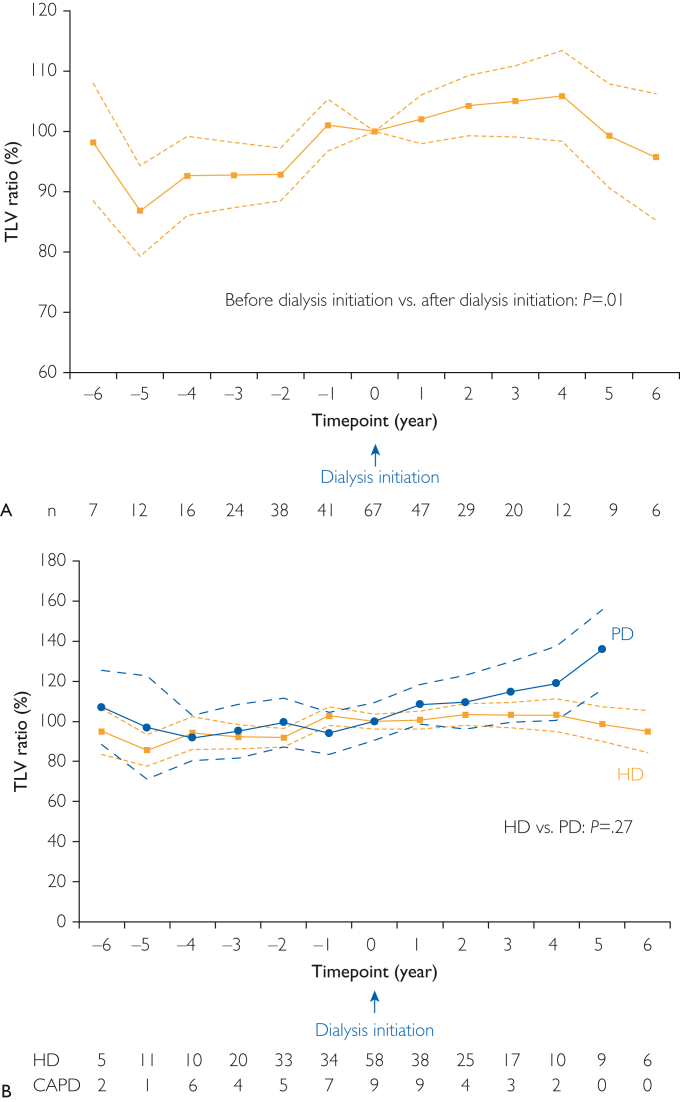
(A) The linear mixed model analysis reported that the least squares mean (95% confidence interval) of the total liver volume ratio at each time point significantly decreased after dialysis initiation (*P*=.01). (B) The linear mixed model analysis reported that the least squares mean (95% confidence interval) of the total liver volume ratio at each time point was lower in patients on HD than in patients on PD, however, it was not statistically significant (*P*=.27). HD, hemodialysis; PD, peritoneal dialysis; TLV, total liver volume.

The changes in the TLV ratio at each time point are shown in Figure 3A. At 6 years before dialysis initiation, the least squares mean TLV ratio was 98.2% (95% CI, 88.4%-108.0%). At 6 years after dialysis initiation, the least squares mean TLV ratio was 95.7% (95% CI, 85.2%-106.2%). The changes in the TLV ratio significantly decreased after dialysis initiation (*P*=.01) (Supplemental Table 4, available online at http://www.mcpiqojournal.org); however, the TLV continued to increase even after dialysis initiation. The actual measured TLV of each patient on HD and PD at each time point is presented in Supplemental Figure 3A and B (available online at http://www.mcpiqojournal.org). The changes in the TLV ratio according to sex are presented in Supplemental Figure 3C. There was no significant difference in the changes in the TLV ratio before and after dialysis initiation between the male and female patients. The changes in the TLV ratio according to TLV quartile are presented in Supplemental Figure 3D. There were significant differences in the changes in the TLV ratio before and after dialysis initiation among the 4 groups.

The univariate linear mixed model analysis revealed that age, serum Alb, serum Creactive protein, and eGFR at dialysis initiation had significant effects on the changes in TLV ratio. The multivariate linear mixed model analysis—using variables that were significant in the univariate analysis—revealed that no predictive variables had significant effects on the changes in the TLV ratio (Table 3). The TLV ratio at each time point, stratified according to HD and PD, are shown in Figure 3B. The TLV ratio after dialysis initiation was higher in the patients on PD than in those on HD; however, it was not statistically significant (*P*=.27) (Supplemental Table 5, available online at http://www.mcpiqojournal.org).

**Table 3:** The Changes (95% Confidence Intervals) in Slope Coefficients of Total Liver Volume Curves by Predictive Variables in Univariate and Multivariable Linear Mixed Model Analyses^a^

	Change in TLV (%/y)								
	Univariate analysis				Multivariate analysis^b^				
	Regression coefficient	SE	Beta	*P* value	Regression coefficient	SE	Beta	*P* value	VIF
Clinical characteristics at dialysis initiation									
Age	−0.33	0.14	−0.317	.02	−0.20	0.15	−0.182	.20	1.140
Male (vs female)	−0.78	3.30	−0.032	.82					
BMI	−0.28	0.51	−0.076	.59					
sBP	−0.04	0.08	−0.074	.60					
dBP	0.14	0.14	0.136	.33					
HR	−0.07	0.14	−0.066	.64					
Hb	0.22	0.86	0.037	.80					
Serum TP	2.40	3.16	0.107	.45					
Serum Alb	12.01	3.68	0.419	.002	5.57	4.95	0.193	.27	1.749
Serum CRP	−1.85	0.76	−0.333	.02	−0.95	0.91	−0.172	.30	1.610
eGFR	−1.93	0.74	−0.345	.01	−1.16	0.79	−0.205	.15	1.148
Serum UN	0.07	0.07	0.145	.31					
HtTKV (per 100 mL)	−0.20	0.10	−0.195	.15					
Mayo classification (per 1 grade)	0.09	1.68	0.007	.96					
HtTLV (per 100 mL)	−0.10	0.10	−0.212	.12					
HtTLV (per 1 category)^c^	−2.49	1.35	−0.245	.07					
PD (vs HD)	8.09	4.31	0.250	.07					

a Alb, albumin; BMI, body mass index; BP, blood pressure; CRP, C-reactive protein; dBP, diastolic blood pressure; eGFR, estimated glomerular filtration rate; Hb, hemoglobin; HD, hemodialysis; HR, heart rate; HtTKV, height-adjusted total kidney volume; HtTLV, height-adjusted total liver volume; PD, peritoneal dialysis; sBP, systolic blood pressure; SE, standard error; TLV, total liver volume; TP, total protein; UN, urea nitrogen; VIF, variance inflation factor.

b The significant variables in the univariate analysis were selected for the multivariate analysis.

c Patients were grouped by HtTLV as follows: quartile 1, <1053.2 mL/m; quartile 2, 1053.2 to <1599.3 mL/m; quartile 3, 1599.3 to <2683.6 mL/m; and quartile 4, ≥2683.6 mL/m.

At 6 years before dialysis initiation, the least squares mean systolic BP value was 126.1 mm Hg (95% CI, 115.2-136.9 mm Hg) (Supplemental Figure 4A, available online at http://www.mcpiqojournal.org). At dialysis initiation, the least squares mean systolic BP was 139.0 mm Hg (95% CI, 135.4-142.6 mm Hg). At 6 years after dialysis initiation, the least squares mean systolic BP value was 137.7 mm Hg (95% CI, 123.3-152.0 mm Hg). The changes in systolic BP did not significantly differ between before and after dialysis initiation (*P*=.17) (Supplemental Table 6, available online at http://www.mcpiqojournal.org). At 4 years after dialysis initiation, the systolic BP was higher in the patients on PD than in those on HD; however, this difference was not statistically significant (*P*=.45) (Supplemental Figure 4B and Supplemental Table 7, available online at http://www.mcpiqojournal.org).

## Discussion

This study revealed that the TKV continued to increase until dialysis initiation; it generally decreased after dialysis initiation. The strongest factor influencing the changes in TKV after dialysis initiation was dialysis style (HD or PD), and TKV was significantly more likely to decrease in the patients on HD than in those on PD. Sex was also a significant factor, and TKV was significantly more likely to decrease in female patients than in male patients. Additionally, TKV in patients with younger age was more likely to decrease after dialysis initiation, although this factor was not statistically significant in the multivariate analysis. To our knowledge, this is the first study to compare changes in TKV between before and after dialysis initiation in patients on HD and those on PD. The results indicated that changes in TKV after dialysis initiation might differ between patients on HD and those on PD. However, the number of patients on PD was small in this study, especially the number of patients who continued PD for 2 or more years, which was only five. The results of this study might be driven by too few patients; therefore, we should be cautious while interpreting the results. However, it may help to reveal factors influencing the progression of PKD and PLD by facilitating comparison of clinical characteristics between patients on HD and those on PD. We hypothesized that higher BP, greater body fluid overload, and lower dialysis efficiency in patients on PD compared with those in patients on HD might have affected the increase in TKV after dialysis initiation in the patients on PD. Indeed, we found that the BP was higher in the patients on PD than in those on HD (Supplemental Figure 4B). Regarding body fluid volume, edema was present in 83.3% of the patients on PD at 1 year after dialysis initiation, whereas it was present in 36.4% of those on HD (Supplemental Table 8, available online at http://www.mcpiqojournal.org). The serum creatinine (Cr) and UN levels were also higher in the patients on PD than in those on HD, implying that the dialysis efficiency was lower and uremia was more serious in the patients on PD than in those on HD. The serum levels of various uremic toxins remain constant in patients on PD, whereas they exhibit saw-tooth-like profiles (ie, with troughs and peaks) in patients on HD.^12^ In this study, the peak concentrations of serum Cr and UN in the patients on HD were lower than those in the patients on PD.

Generally, body fluid overload is a common problem in patients on PD.^13^ In the European Body Composition Monitoring study, which involved 639 patients on PD in 6 European countries, Van Biesen et al^14^ found that severe fluid overload was present in 25.2% of the study population. Regarding hydration status, as measured using the bioimpedance analysis method, Devolder et al^15^ and Yılmaz et al^16^ noted that the ratio of overhydration to extracellular water was higher in patients on PD than in those on HD. Thus, patients on PD may be more likely to experience body fluid overload than those on HD. This is presumably because HD provides easier and more efficient control of extracellular volume overload.^17^ Furthermore, physicians may evaluate fluid status less frequently in patients on PD than in those on HD.^18^ Body fluid overload causes various symptoms related to abnormal fluid storage, such as peripheral edema, pleural effusion, and body cavity effusion.^19^ These symptoms are related to elevated mean circulatory filling pressure, which causes higher capillary pressures; increased capillary pressure promotes fluid movement to the interstitial space and into renal or hepatic cysts. This presumptive mechanism may support our hypothesis that volume overload can increase TKV. We did not report enough evidence that fluid overload was more frequent in patients on PD than in those on HD in this study. However, fluid overload could be a reason why the average TKV or TLV continued to increase after dialysis initiation in the patients on PD. Future studies to evaluate body fluid in detail and investigate the relationship between body fluid and cyst growth in ADPKD are necessary. If our hypothesis is correct, high water intake should be carefully monitored in patients with ADPKD not on dialysis because excess intake may lead to body fluid overload, although high water intake is generally recommended for patients with ADPKD not on dialysis.

Yamamoto et al^20^ reported that the TKV decreased after kidney transplantation. A greater reduction in TKV in patients with better posttransplantation renal function may be caused by more efficient elimination of the effects of uremia on tubular epithelium proliferation, greater reduction in blood flow to native kidneys, and improvement of fluid overload^8^; these changes would support our hypothesis.

We previously reported that renal-TAE can reduce the renal volume in patients with ADPKD.^21^ This reduction is presumably caused by acute renal infarction related to severe renal artery occlusion. However, a small amount of renal artery flow or recanalization of the bilateral renal arterial branches remains in some patients after renal-TAE.^22^ Thus, renal-TAE may not completely shut down the renal artery; it may simply reduce renal blood flow. However, the kidney volume consistently decreases after renal-TAE,^21^ possibly through a process that occurs after atherosclerotic renal artery stenosis.^23^ Thus, decreases in renal artery flow cause reductions in TKV. Cardiac output is generally correlated with mean arterial pressure; renal blood flow constitutes 20%-25% of cardiac output and might also be correlated with mean arterial pressure. Furthermore, BP is correlated with renal artery blood flow, which may explain why the kidney volume decreases because of lower BP after dialysis initiation, particularly in patients on HD.

We should also consider the effectiveness of acquired cystic disease of the kidney (ACDK). This is because ACDK has been reported in 7%-22% of people with chronic kidney disease, who have already acquired cystic kidney disease before starting dialysis.^24^ Almost 60% of people on dialysis for 2-4 years develop ACDK, and approximately 90% of people on dialysis for 8 years develop ACDK. Therefore, ACDK might have been present in some enrolled patients in this study, especially in patients with a long duration of dialysis.^24^ However, the size of the kidneys in people with ACDK is usually normal or smaller within 6 years after dialysis initiation compared with the predialysis size,^25^ and the effect of dialysis on TKV is considered small in these patients. Additionally, it is difficult to differentiate between renal cysts due to ADPKD and those due to ACDK using CT. Therefore, we did not differentiate cysts due to ADPKD and those due to ACDK in this study. The kidney size in patients with ACDK generally decreases after kidney transplantation compared with the pretransplantation size, which is considered a result of improvement of uremia.^26^ Therefore, the mechanisms underlying ACDK and PKD may be similar, and there may be a common pathway for cysts to grow in ADPKD and ACDK. This may be a reason why the TKV decreased more than TLV; ie, by improving uremia after dialysis initiation.

In our previous study, younger age had a significant association with increased renal volume reduction rate after renal-TAE; TKV was more likely to decrease in younger patients.^21^ We presumed that renal cyst walls become stiff in older patients. In the present study, TKV and TLV were more likely to decrease in younger patients, although age was not a significant factor in the multivariate analysis. Yamamoto et al^20^ also reported that TKV was more likely to decrease after kidney transplantation in younger patients. The previous and present results may have similar causes.

The changes in the slope coefficients of TKV curves were larger in the male patients than in the female patients in the multivariate analysis. The TKV tended to continue to increase in the male patients compared with that in the female patients after dialysis initiation. Generally, male sex is considered a prognostic factor for PKD progression,^27^ which may have been the reason for the continued increase in TKV in the male patients in our study. Another reason might be that the rate of patients on PD was higher in male patients than that in female patients in our study (17.9% vs 7.5%, respectively).

Changes in TLV and TKV may be influenced by similar factors. Notably, PD had a strong effect on the changes in TLV after dialysis initiation, although the relationship was not statistically significant. This result was consistent with our previous finding that rigorous BP control and improvement of interstitial tissue edema may reduce TLV.^28^ In contrast to TKV, we found that the TLV continued to increase even after dialysis initiation, although the increases in TLV decreased after dialysis initiation. Yamamoto et al^20^ also reported that the TLV did not decrease after kidney transplantation. Importantly, hepatic artery pressure may be regulated to maintain constant hepatic blood flow,^29^ whereas renal arteries originate from the abdominal artery; blood flow to the liver is unique because of dual supply from the portal vein and hepatic artery. Accordingly, the changes in blood flow related to changes in systemic BP after dialysis initiation are smaller in the liver than in the kidney. Additionally, PLD is more common in women than in men, presumably because of estrogen-related effects.^30^, ^31^, ^32^ Some studies have reported that multiple pregnancies and exogenous estrogen treatment are risk factors for the growth of hepatic cysts,^33^^,^^34^ and premenopausal status may be an independent risk factor for polycystic liver growth.^35^ Thus, factors that influence changes in PLD are more complex than factors that influence changes in PKD; this complexity may contribute to the reduced likelihood of decreases in TLV after dialysis initiation and kidney transplantation compared with that of decreases in TKV. This area requires further investigation.

This study had some limitations. First, the number of enrolled patients was not large, and the study used a single-center, retrospective design. In particular, the number of patients on PD was small; therefore, it may be difficult to generalize the results of this study. Second, there were comparatively few images available for each time point. Third, all the enrolled patients were of Japanese ethnicity, which may reduce the generalizability of the findings. Fourth, not all the patients underwent genetic testing because this is not usually available in Japan. Therefore, we could not assess the effectiveness of genetic types. However, this study had some strengths. To our knowledge, this is the largest study to investigate the changes in TKV and TLV among patients with ADPKD who began receiving dialysis. We used long observation periods before and after dialysis initiation (6 years); we also analyzed the changes in TKV and TLV using linear mixed models. Importantly, we observed significant changes in TKV before and after dialysis initiation; the multivariate analysis reported that PD had the strongest effect on the changes in TKV after dialysis initiation. Furthermore, TKV and TLV were measured by a single researcher using validated specific software, which minimized the measurement error.

In conclusion, this study revealed that the TKV continued to increase until dialysis initiation and then generally decreased after dialysis initiation; however, the TLV continued to increase even after dialysis initiation. The increases in TKV and TLV were greater in patients on PD than in patients on HD, presumably because of higher BP, greater body fluid overload, and lower dialysis efficiency.

## Potential Competing Interests

The authors report no competing interests.
