# Supplementary material for: Changes in Kidney and Liver Volumes in Patients With Autosomal Dominant Polycystic Kidney Disease Before and After Dialysis Initiation
Source: Mayo Clin Proc Innov Qual Outcomes. 2023 Jan 20;7(1):69–80. doi: 10.1016/j.mayocpiqo.2022.12.005 (PMC9873948; doi:10.1016/j.mayocpiqo.2022.12.005)
Supplement: Supplemental Tables [file mmc1.doc]

**Supplemental Tables: Changes in kidney and liver volume in patients with polycystic kidney disease before and after dialysis initiation**

**(Supplemental Table 1)** Diagnostic criteria for autosomal dominant polycystic kidney disease proposed by Progressive Renal Disease Research (Ministry of Health, Labour and Welfare of Japan), presented in clinical practice guidelines for autosomal dominant polycystic kidney disease (2nd edition).

For individuals with a family history

1. At least three cysts in both kidneys detected by ultrasound.
2. At least five cysts in both kidneys detected by CT or MRI.

For individuals without a family history

1) At least three cysts in both kidneys detected by ultrasound, CT, or MRI in individuals aged 15 years or younger, in addition to exclusion of the diseases listed below.

2) At least five cysts in both kidneys detected by ultrasound, CT or MRI in individuals aged 16 years or older, in addition to exclusion of the diseases listed below.

Diseases to be excluded

multiple simple renal cysts, renal tubular acidosis, multicystic kidney (multicystic dysplastic kidney), multilocular cysts of the kidney, medullary cystic disease of the kidney (juvenile nephronophthisis), acquired cystic disease of the kidney, and autosomal recessive polycystic kidney disease

**(Supplemental Table 2)** Linear mixed model analysis to estimate the changes of slope coefficients of TKV ratio curves

Linear mixed model analysis (with the patient as a variable factor) was performed, considering the interaction between the timing of dialysis initiation and time to estimate the changes in slope coefficients of TKV ratio.

|  | Numerator degree of freedom | Denominator degree of freedom | *F value* | *P value* |
| --- | --- | --- | --- | --- |
| Intercept | 1 | 213.60 | 2809.46 | <.001 |
| Dialysis initiation | 1 | 445.25 | 32.90 | <.001 |
| Time | 1 | 421.30 | 5.79 | .01 |
| Time*Dialysis initiation | 1 | 444.35 | 45.65 | <.001 |

**(Supplemental Table 3)** Linear mixed model analysis to estimate the changes of slope coefficients of TKV ratio curves

Linear mixed model analysis (with the patient as a variable factor) was performed, considering the interaction between dialysis style (HD or PD) and time to estimate the changes in slope coefficients of TKV ratio curves.

|  | Numerator degree of freedom | Denominator degree of freedom | *F value* | *P value* |
| --- | --- | --- | --- | --- |
| Intercept | 1 | 125.799 | 1639.58 | <.001 |
| Dialysis style (HD or PD) | 1 | 123.384 | 20.42 | <.001 |
| Time | 12 | 375.192 | 21.72 | <.001 |
| Time*Dialysis style | 11 | 374.459 | 18.53 | <.001 |

**(Supplemental Table 4)** Linear mixed model analysis to estimate the changes of slope coefficients of TLV ratio curves

Linear mixed model analysis (with the patient as a variable factor) was performed, considering the interaction between the timing of dialysis initiation and time to estimate the changes in slope coefficients of TLV ratio.

|  | Numerator degree of freedom | Denominator degree of freedom | *F value* | *P value* |
| --- | --- | --- | --- | --- |
| Intercept | 1 | 139.65 | 2809.88 | <.001 |
| Dialysis initiation | 1 | 303.82 | 5.20 | .02 |
| Time | 1 | 282.95 | 1.97 | .16 |
| Time*Dialysis initiation | 1 | 307.02 | 6.45 | .01 |

**(Supplemental Table 5)** Linear mixed model analysis to estimate the changes of slope coefficients of TLV ratio curves

Linear mixed model analysis (with the patient as a variable factor) was performed, considering the interaction between dialysis style (HD or CAPD) and time to estimate the changes in slope coefficients of TLV ratio curves.

|  | Numerator degree of freedom | Denominator degree of freedom | *F value* | *P value* |
| --- | --- | --- | --- | --- |
| Intercept | 1 | 78.796 | 3043.81 | <.001 |
| Dialysis style (HD or PD) | 1 | 76.365 | 2.57 | .11 |
| Time | 12 | 255.513 | 2.40 | .006 |
| Time*Dialysis style | 11 | 254.523 | 1.24 | .27 |

**(Supplemental Table 6)** Linear mixed model analysis to estimate the changes of slope coefficients of systolic blood pressure curves

Linear mixed model analysis (with the patient as a variable factor) was performed, considering the interaction between the timing of dialysis initiation and time to estimate the changes in slope coefficients of systolic blood pressure curves.

|  | Numerator degree of freedom | Denominator degree of freedom | *F value* | *P value* |
| --- | --- | --- | --- | --- |
| Intercept | 1 | 267.22 | 6009.14 | <.001 |
| Dialysis initiation | 1 | 363.28 | 3.46 | 0.06 |
| Time | 1 | 340.10 | 2.69 | 0.10 |
| Time*Dialysis initiation | 1 | 364.45 | 1.94 | 0.17 |

**(Supplemental Table 7)** Linear mixed model analysis to estimate the changes of slope coefficients of systolic blood pressure curves

Linear mixed model analysis (with the patient as a variable factor) was performed, considering the interaction between dialysis style (HD or CAPD) and time to estimate the changes in slope coefficients of systolic blood pressure curves.

|  | Numerator degree of freedom | Denominator degree of freedom | *F value* | *P value* |
| --- | --- | --- | --- | --- |
| Intercept | 1 | 124.098 | 3580.04 | <.001 |
| Dialysis style (HD or PD) | 1 | 125.597 | 0.34 | .56 |
| Time | 12 | 305.946 | 0.86 | .59 |
| Time*Dialysis style | 11 | 304.382 | 1.00 | .45 |

**(Supplemental Table 8)** Comparison of clinical characteristics of patients on HD between patients on PD

|  | Serum Cr ^a^ | | | Serum UN ^b^ | | | Leg edema ^c^ | | |
| --- | --- | --- | --- | --- | --- | --- | --- | --- | --- |
|  | HD^†^ | PD | *P value* | HD^†^ | PD | *P value* | HD | PD | *P value* |
| Dialysis initiation | 7.3 ± 2.7  (n=85) | 8.8 ± 3.7  (n=10) | .12 | 75.8 ± 24.8  (n=85) | 76.8 ± 27.5  (n=10) | .91 | 70.2% (59/84)  (n=84) | 66.7% (6/9)  (n=9) | .83 |
| 1 year after | 8.3±2.5  (n=55) | 10.5±5.3  (n=9) | .05 | 57.9±14.9  (n=55) | 67.5±14.6  (n=9) | .09 | 36.4% (12/33)  (n=33) | 83.3% (5/6)  (n=6) | .03 |
| 2 years after | 8.7±2.4  (n=33) | 12.0±6.7  (n=5) | .04 | 52.7±12.0  (n=33) | 69.1±20.6  (n=5) | .01 | 39.1% (9/23)  (n=23) | 75.0% (3/4)  (n=4) | .18 |
| 3 years after | 9.4±2.5  (n=23) | 11.9±4.8  (n=3) | .15 | 53.3±15.1  (n=23) | 66.3±21.7  (n=3) | .19 | 33.3% (4/12)  (n=12) | 100% (2/2)  (n=2) | .05 |

^a^ Patients who did not take blood tests were excluded.

^b^ Blood test performed at the beginning of hemodialysis.

^c^ Patients whose leg edema were unknown (no description about leg edema in medical records) were excluded.
