# Supplementary material for: Changes in Kidney and Liver Volumes in Patients With Autosomal Dominant Polycystic Kidney Disease Before and After Dialysis Initiation
Source: Mayo Clin Proc Innov Qual Outcomes. 2023 Jan 20;7(1):69–80. doi: 10.1016/j.mayocpiqo.2022.12.005 (PMC9873948; doi:10.1016/j.mayocpiqo.2022.12.005)
Supplement: Supplemental Figure [file mmc2.pptx]

## Slide 1
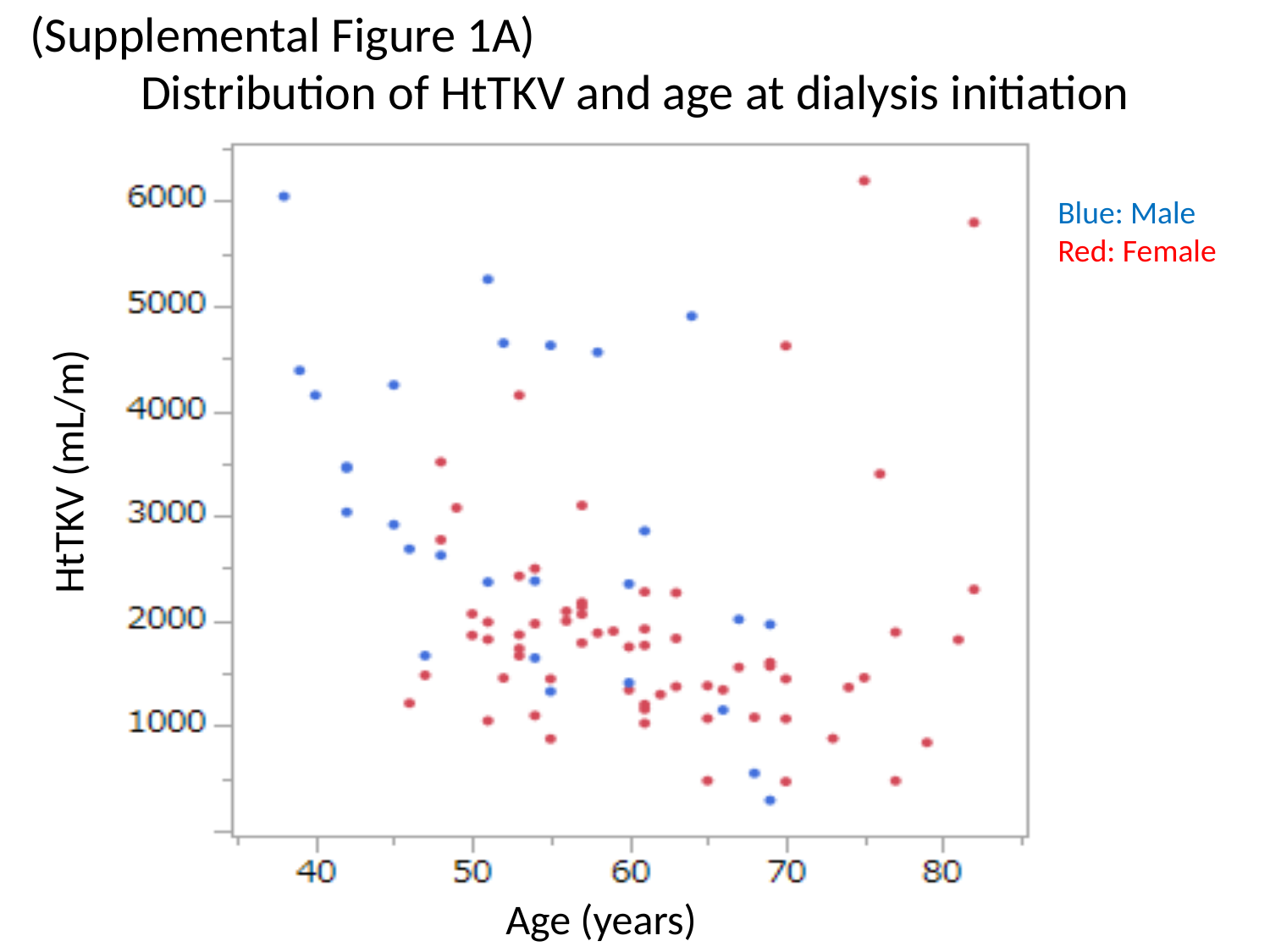

(Supplemental Figure 1A)
# Distribution of HtTKV and age at dialysis initiation
Blue: Male
Red: Female
HtTKV (mL/m)
Age (years)

## Slide 2
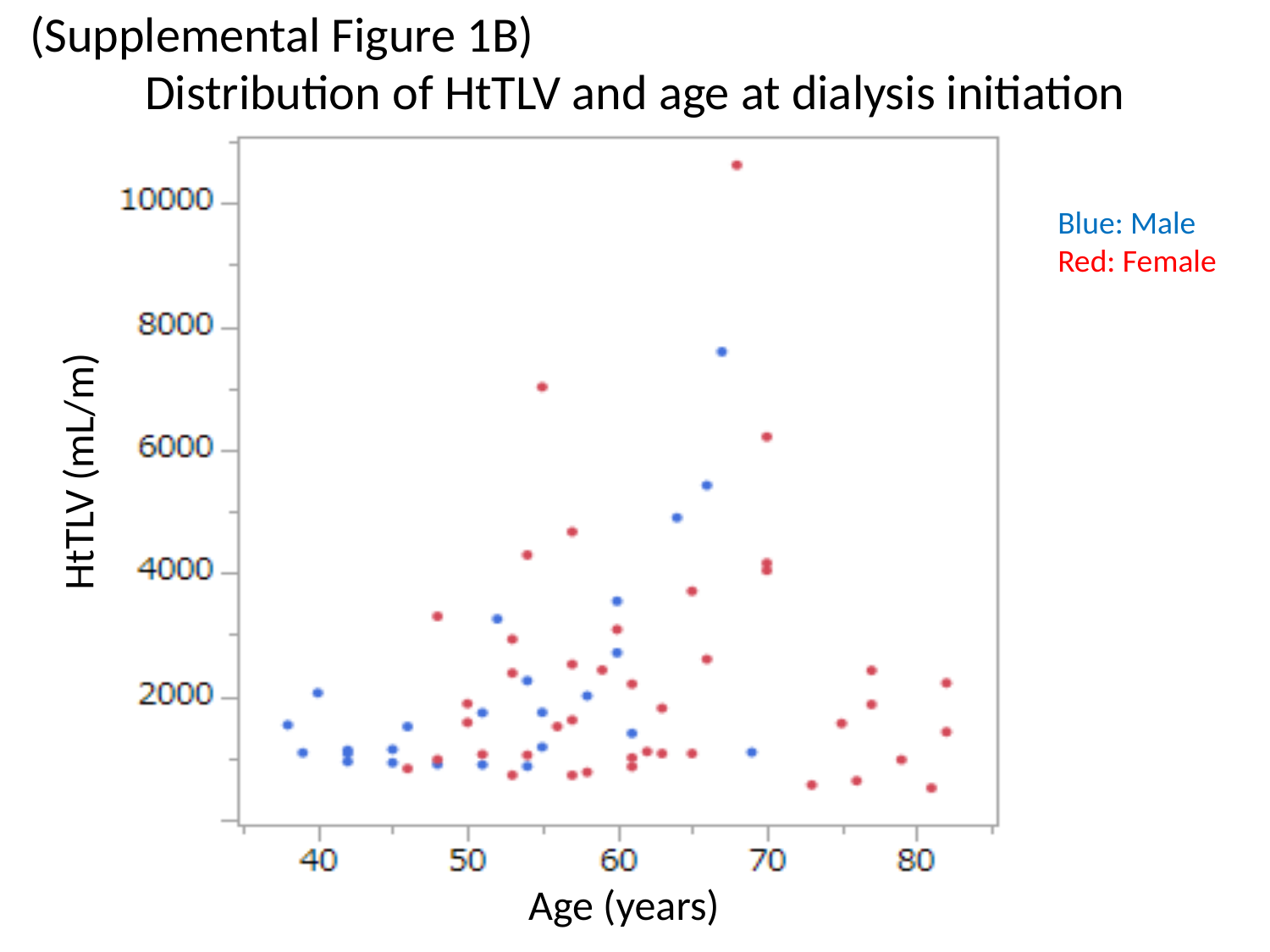

(Supplemental Figure 1B)
# Distribution of HtTLV and age at dialysis initiation
Blue: Male
Red: Female
HtTLV (mL/m)
Age (years)

## Slide 3
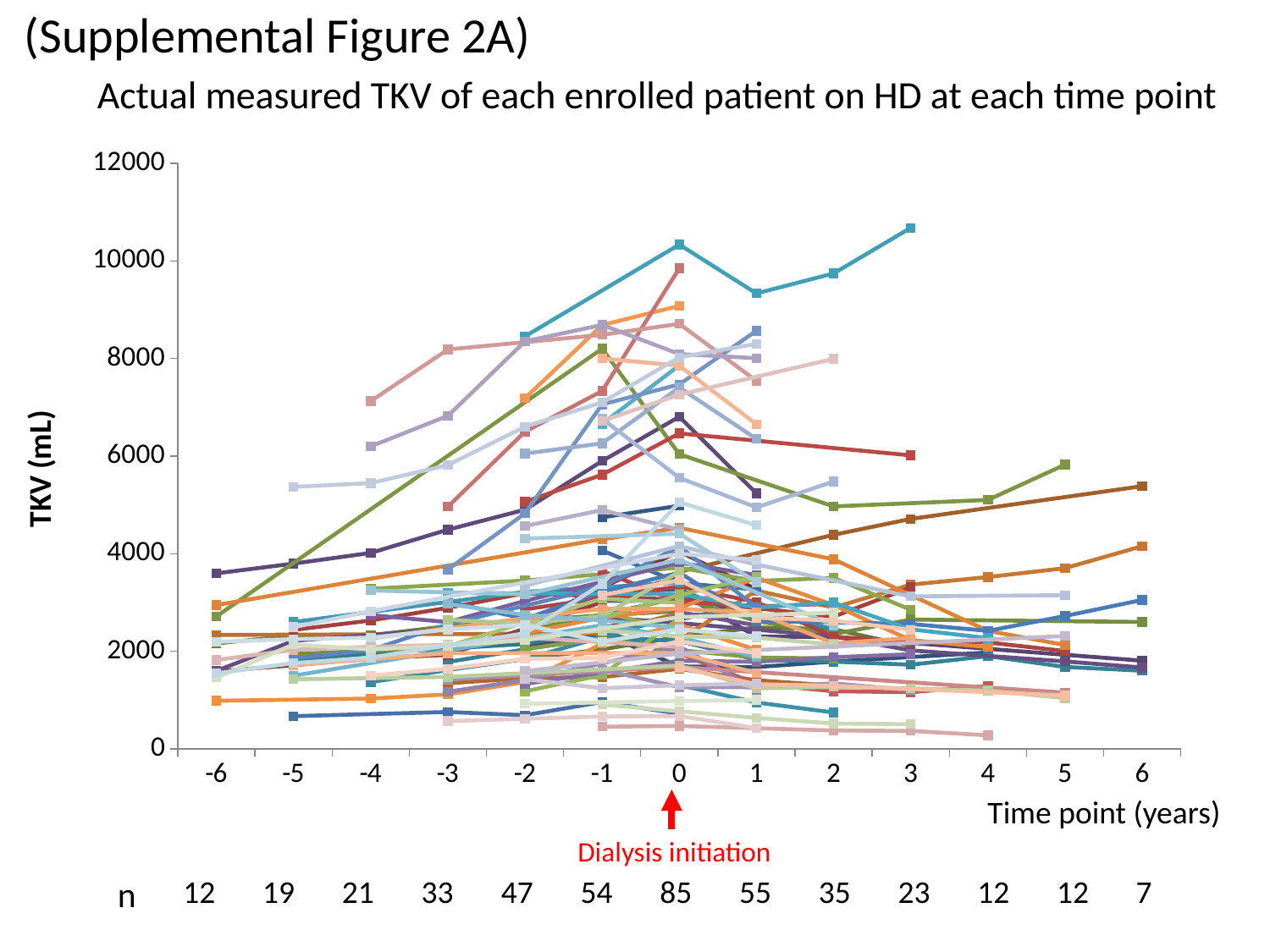

(Supplemental Figure 2A)
Actual measured TKV of each enrolled patient on HD at each time point
### Chart
| Category | | | | | | | | | | | | | | | | | | | | | | | | | | | | | | | | | | | | | | | | | | | | | | | | | | | | | | | | | | | | | | | | | | | | | | | | | | | | | | | | | | | | | | | | | | | | | | | |
|---|---|---|---|---|---|---|---|---|---|---|---|---|---|---|---|---|---|---|---|---|---|---|---|---|---|---|---|---|---|---|---|---|---|---|---|---|---|---|---|---|---|---|---|---|---|---|---|---|---|---|---|---|---|---|---|---|---|---|---|---|---|---|---|---|---|---|---|---|---|---|---|---|---|---|---|---|---|---|---|---|---|---|---|---|---|---|---|---|---|---|---|---|---|---|---|
| -6 | None | None | None | None | None | None | None | None | 2161.681 | None | None | None | None | None | None | 3597.61 | None | 2335.204 | None | 1589.496 | 2714.902 | 1599.28 | None | None | None | None | None | None | None | None | None | None | None | None | None | 2948.381 | None | None | None | None | None | None | None | None | None | None | None | 986.658 | None | None | None | None | None | None | None | None | None | None | None | None | None | None | None | None | None | None | None | None | None | None | None | None | None | None | None | None | None | None | None | 1823.099 | None | None | None | None | None | None | 1460.13 | None | 1548.22 | None | None | None | None | None | 2195.07 |
| -5 | None | None | None | None | None | None | None | None | 2319.229 | None | 1782.566 | None | None | None | 1943.411 | 3797.316 | None | None | None | 1714.143 | None | 2214.839 | None | None | None | 2438.561 | None | None | None | None | 671.23 | None | None | None | 2600.217 | None | None | None | None | None | None | None | None | None | None | None | None | None | 1853.19 | None | None | None | None | None | None | None | None | None | 1501.93 | None | None | None | None | None | None | 1718.66 | None | None | 1427.31 | None | None | None | None | None | None | None | None | None | None | 2031.122 | None | None | None | None | 5370.3 | None | 2138.25 | None | 1758.92 | None | 2494.23 | None | None | None | 2248.83 |
| -4 | None | None | None | None | None | None | None | None | 2329.126 | None | 1958.901 | None | None | None | None | 4016.225 | None | None | None | 1857.474 | None | None | 1367.928 | None | None | 2631.814 | None | None | None | None | None | None | 3281.947 | None | None | None | None | None | None | None | None | None | None | None | None | 2742.87 | None | 1031.21 | 2025.58 | None | None | None | None | None | None | None | None | None | None | 2265.06 | None | None | None | None | None | None | None | 7126.51 | None | 6200.61 | 3246.93 | None | None | None | None | None | None | None | None | 2090.8 | None | None | None | None | 5445.4 | None | 2039.6 | None | 1872.52 | 1503.17 | 2821.79 | None | None | None | 2279.21 |
| -3 | None | None | None | None | None | None | None | None | 2515.448 | None | 2056.589 | 1345.452 | None | None | 2079.008 | 4492.802 | 1781.725 | None | None | 1927.677 | None | None | 1616.204 | None | None | 2892.4 | None | None | None | None | 757.457 | None | None | None | None | None | None | None | None | None | None | None | 3015.65 | None | None | 2597.92 | 3194.59 | 1118.09 | 2554.34 | None | None | None | None | None | 3676.65 | 4963.44 | 2102.52 | 1172.72 | None | None | None | 1425.07 | 2646.7 | 1428.11 | 2968.58 | 1962.71 | None | 8185.27 | 1475.05 | 6825.52 | None | None | None | None | None | None | None | None | None | 2132.17 | None | None | None | None | 5816.81 | None | 2113.38 | None | None | 1640.28 | None | 570.0 | None | None | 2447.31 |
| -2 | 2087.549 | 2571.777 | 1842.243 | 2038.758 | None | None | None | None | None | None | 2151.706 | 1448.451 | 3172.885 | None | None | 4901.934 | None | None | None | None | None | None | 1842.57 | None | None | 3191.58 | 2047.19 | None | None | None | 690.46 | 2859.18 | 3449.93 | None | 3217.49 | None | None | None | None | None | 8454.882 | 2374.21 | 2667.22 | 5062.26 | 1180.49 | 3032.77 | None | 1370.95 | None | None | None | 1331.01 | None | 7187.02 | 4827.21 | 6502.18 | None | 1408.98 | None | 2678.65 | None | None | 2566.67 | None | 2748.09 | None | 6052.62 | None | None | 8351.88 | 3168.76 | None | None | None | 2279.163 | 4568.189 | 4310.124 | None | 3346.072 | 2282.88 | None | 1595.174 | None | None | 6604.06 | None | 2228.9 | 1429.9 | 2370.1 | 1846.24 | None | 618.06 | 923.97 | None | 2544.86 |
| -1 | 2370.212 | None | 2040.563 | 2500.371 | None | None | 4745.635 | None | 2737.67 | None | 2413.439 | 1472.491 | None | None | None | 5899.94 | 2296.88 | 2365.71 | None | None | 8200.79 | 2698.96 | None | None | 4068.03 | None | 2258.57 | 3034.54 | None | None | 960.76 | None | None | None | None | 4295.46 | None | 3639.43 | 2807.21 | None | None | 2691.55 | 3215.85 | 5618.94 | 1515.3 | None | None | 2155.11 | 3315.57 | 1951.69 | 2679.96 | None | 6661.745 | 8686.28 | 7060.0 | 7334.5 | 3079.26 | None | 2534.31 | 2870.95 | None | None | 2721.61 | 1625.42 | 2634.64 | None | 6262.53 | 8485.79 | None | 8688.96 | None | 7999.713 | 6766.996 | 454.809 | 2429.204 | 4893.564 | None | None | None | None | 908.68 | None | None | 3151.25 | 7101.95 | 6721.35 | None | 1245.05 | 3384.34 | 1863.77 | None | 664.67 | None | None | 2198.51 |
| 0 | 1691.29 | 4043.427 | 2296.969 | 2654.049 | None | 3629.079 | 4982.705 | None | 2822.97 | 3206.82 | 1916.33 | 1640.78 | 3267.11 | None | 3094.65 | 6806.35 | 2209.06 | 2786.62 | None | 3550.47 | 6039.51 | 2578.55 | 2739.28 | 2232.42 | 3398.47 | 3056.06 | 2024.35 | 3038.22 | 1310.28 | None | 721.95 | 3330.5 | 3714.48 | 2801.73 | 3407.69 | 4530.62 | None | 3013.14 | 2410.26 | None | 10334.727 | 2882.85 | 3613.63 | 6466.01 | 2675.89 | 3817.09 | 3171.4 | 2544.46 | 4152.28 | 1958.79 | 3152.63 | 1806.13 | 7858.85 | 9079.77 | 7470.2 | 9847.15 | 2991.67 | 2099.81 | 2289.64 | 2868.88 | None | 1679.82 | 3629.1 | 1264.78 | 2479.35 | 1962.99 | 7406.91 | 8710.72 | 1709.46 | 8091.38 | 3877.58 | 7853.534 | 5553.601 | 471.648 | 2304.91 | 4489.819 | 4409.324 | 1665.379 | 4156.525 | 2092.13 | 771.49 | 1954.302 | None | 3456.78 | 8025.15 | 7260.39 | 2701.77 | 1304.89 | 5053.7 | 2204.36 | 3999.23 | 674.52 | 981.57 | None | 2450.78 |
| 1 | 1677.435 | 3270.76 | 2488.1 | 2982.922 | None | None | None | None | None | 2303.65 | None | 1407.33 | 2622.64 | None | 2621.87 | 5235.25 | 1825.08 | None | None | None | None | None | 2781.62 | 3243.8 | 3266.8 | None | 1873.12 | None | 951.48 | None | None | 3009.87 | 3441.59 | 2533.52 | None | None | None | 2917.67 | None | None | 9332.796 | 3508.49 | 2620.74 | None | None | 3562.77 | 2897.55 | 2028.58 | 2895.7 | 1331.19 | 3528.05 | 1784.9 | None | None | 8568.96 | None | None | 1212.05 | 1884.7 | 2793.61 | None | None | None | 1263.26 | None | 1557.31 | 6352.42 | 7539.97 | 1241.79 | 8007.65 | None | 6655.067 | 4948.658 | None | 2280.395 | None | 3417.847 | 1326.898 | 3778.295 | None | 635.15 | None | None | 2690.77 | 8301.34 | None | None | 1340.82 | 4582.61 | 1961.41 | 3875.4 | 427.84 | 1002.95 | None | 2306.16 |
| 2 | 1789.227 | None | 2453.082 | None | None | 4386.74 | None | None | None | 2294.36 | None | 1296.44 | None | None | 2353.82 | None | 1788.56 | None | None | None | 4968.23 | 2307.28 | 2458.59 | 2895.78 | None | 2710.25 | 1854.11 | None | 747.38 | None | None | 2299.01 | 3504.17 | None | None | 3884.8 | None | 2976.14 | None | None | 9743.854 | 2946.49 | None | None | None | None | 2997.7 | None | None | 1181.87 | None | 1878.85 | None | None | None | None | None | None | None | 2163.97 | None | None | None | 1342.79 | None | None | None | None | 1270.4 | None | 2533.24 | None | 5478.181 | 376.96 | 2139.832 | None | None | 1293.098 | None | None | 520.92 | None | None | 2628.6 | None | 7994.4 | 2789.74 | None | None | None | None | None | None | None | None |
| 3 | None | None | 2103.07 | None | None | 4712.73 | None | None | None | None | None | None | None | None | 2650.2 | None | 1725.4 | None | None | None | None | 2019.07 | None | 3366.31 | None | 3308.53 | None | None | None | None | None | 2192.96 | 2851.69 | None | None | None | None | None | None | None | 10677.24 | 2229.95 | 2560.93 | 6016.06 | None | None | 2451.0 | None | None | 1162.26 | None | 1940.04 | None | None | None | None | None | None | None | 2271.44 | None | None | None | 1202.21 | None | None | None | None | 1239.99 | None | None | None | None | 369.235 | None | None | None | None | 3125.03 | None | 507.56 | None | None | 2441.02 | None | None | None | None | None | None | None | None | None | None | None |
| 4 | 1975.44 | None | None | None | None | None | None | None | None | None | None | None | None | None | None | None | 1901.0 | None | None | None | 5101.09 | None | None | 3521.92 | None | None | None | None | None | None | None | 2182.49 | None | None | None | 2418.57 | None | None | None | None | None | 2086.08 | 2419.42 | None | None | None | 2271.1 | None | None | 1291.71 | None | None | None | None | None | None | None | None | None | None | None | None | None | None | None | None | None | None | 1194.47 | None | None | None | None | 279.545 | None | None | None | None | None | None | None | None | None | None | None | None | None | None | None | None | None | None | None | None | None |
| 5 | None | None | None | None | None | None | None | None | None | None | None | None | None | None | None | None | 1680.15 | None | None | None | 5826.37 | 1793.01 | None | 3703.68 | None | None | None | None | None | None | None | 2004.09 | None | None | None | 2129.36 | None | None | None | None | None | None | 2724.3 | None | None | None | None | None | None | None | None | None | None | None | None | None | None | None | None | None | None | 1153.36 | None | None | None | None | None | None | 1043.04 | None | None | None | None | None | None | None | None | 1095.9 | 3150.33 | None | None | 2313.51 | None | None | None | None | None | None | None | None | None | None | None | None | None |
| 6 | None | None | None | None | None | 5383.96 | None | None | None | 1807.29 | None | None | None | None | 2600.86 | None | 1602.84 | None | None | None | None | 1667.23 | None | 4154.7 | None | None | None | None | None | None | None | None | None | None | None | None | None | None | None | None | None | None | 3052.41 | None | None | None | None | None | None | None | None | None | None | None | None | None | None | None | None | None | None | None | None | None | None | None | None | None | None | None | None | None | None | None | None | None | None | None | None | None | None | None | None | None | None | None | None | None | None | None | None | None | None | None | None |Time point (years)
Dialysis initiation
n
12
19
21
33
47
54
85
55
35
23
12
12
7

## Slide 4
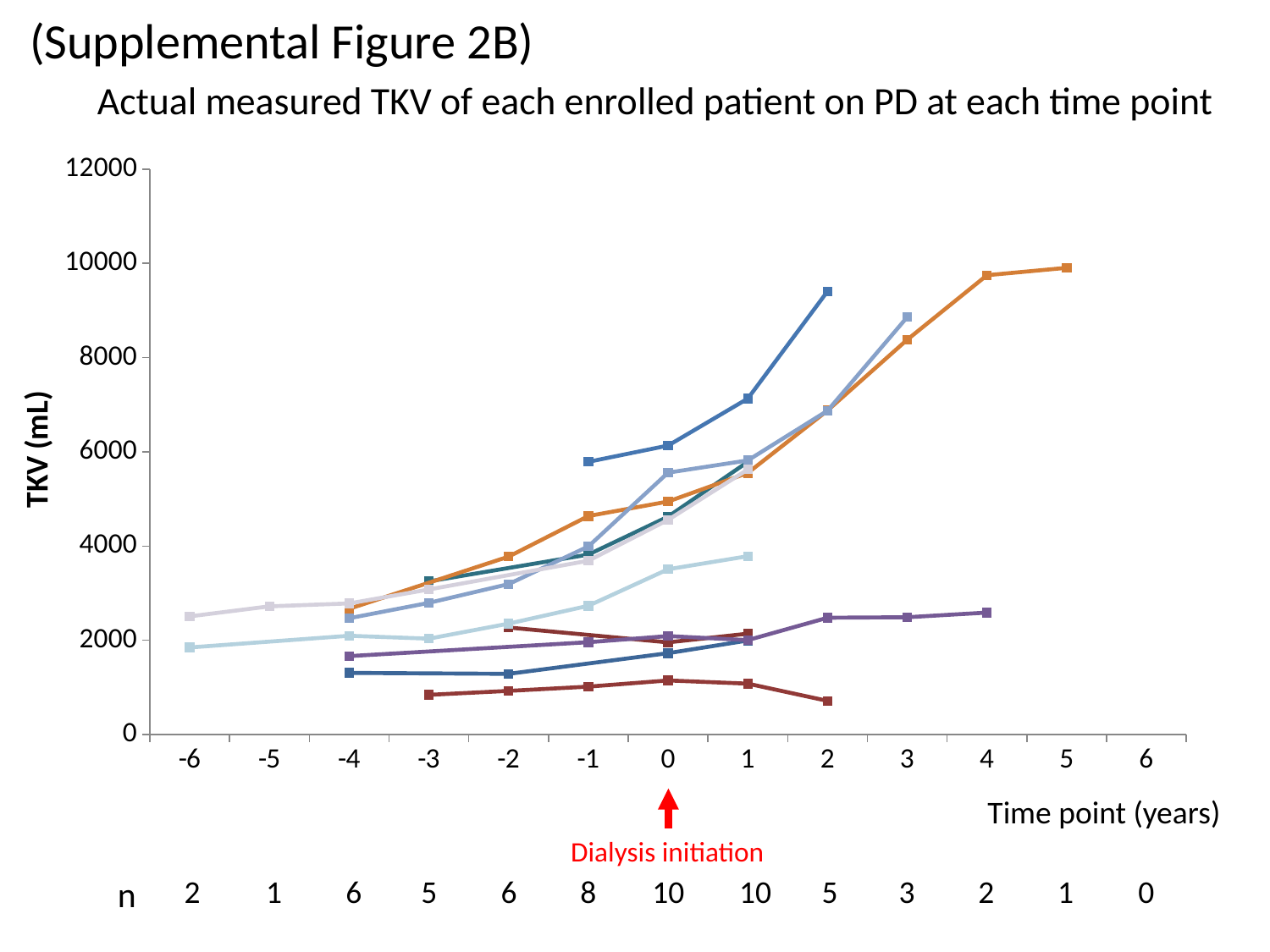

(Supplemental Figure 2B)
Actual measured TKV of each enrolled patient on PD at each time point
### Chart
| Category | | | | | | | | | | | | | | | | | | | | | | | | | | | | | | | | | | | | | | | | | | | | | | | | | | | | | | | | | | | | | | | | | | | | | | | | | | | | | | | | | | | | | | | | | | | | | | | |
|---|---|---|---|---|---|---|---|---|---|---|---|---|---|---|---|---|---|---|---|---|---|---|---|---|---|---|---|---|---|---|---|---|---|---|---|---|---|---|---|---|---|---|---|---|---|---|---|---|---|---|---|---|---|---|---|---|---|---|---|---|---|---|---|---|---|---|---|---|---|---|---|---|---|---|---|---|---|---|---|---|---|---|---|---|---|---|---|---|---|---|---|---|---|---|---|
| -6 | None | None | None | None | None | None | None | None | None | None | None | None | None | None | None | None | None | None | None | None | None | None | None | None | None | None | None | None | None | None | None | None | None | None | None | None | None | None | None | None | None | None | None | None | None | None | None | None | None | None | None | None | None | None | None | None | None | None | None | None | None | None | None | None | None | None | None | None | None | None | None | None | None | None | None | None | None | None | None | None | None | None | 1847.3 | None | None | None | None | None | None | None | None | None | None | 2507.92 | None |
| -5 | None | None | None | None | None | None | None | None | None | None | None | None | None | None | None | None | None | None | None | None | None | None | None | None | None | None | None | None | None | None | None | None | None | None | None | None | None | None | None | None | None | None | None | None | None | None | None | None | None | None | None | None | None | None | None | None | None | None | None | None | None | None | None | None | None | None | None | None | None | None | None | None | None | None | None | None | None | None | None | None | None | None | None | None | None | None | None | None | None | None | None | None | None | 2720.3 | None |
| -4 | None | None | None | None | None | None | None | None | None | None | None | None | None | None | None | None | None | None | 1308.306 | None | None | None | None | None | None | None | None | None | None | 2668.872 | None | None | None | None | None | None | None | None | None | 1663.911 | None | None | None | None | None | None | None | None | None | None | None | None | None | None | None | None | None | None | None | None | 2468.6 | None | None | None | None | None | None | None | None | None | None | None | None | None | None | None | None | None | None | None | None | None | 2096.41 | None | None | None | None | None | None | None | None | None | None | 2782.09 | None |
| -3 | None | None | None | None | 3249.381 | None | None | None | None | None | None | None | None | 842.312 | None | None | None | None | None | None | None | None | None | None | None | None | None | None | None | None | None | None | None | None | None | None | None | None | None | None | None | None | None | None | None | None | None | None | None | None | None | None | None | None | None | None | None | None | None | None | 2794.99 | None | None | None | None | None | None | None | None | None | None | None | None | None | None | None | None | None | None | None | None | None | 2033.96 | None | None | None | None | None | None | None | None | None | None | 3079.1 | None |
| -2 | None | None | None | None | None | None | None | 2270.978 | None | None | None | None | None | 926.226 | None | None | None | None | 1288.43 | None | None | None | None | None | None | None | None | None | None | 3775.93 | None | None | None | None | None | None | None | None | None | None | None | None | None | None | None | None | None | None | None | None | None | None | None | None | None | None | None | None | None | None | 3192.16 | None | None | None | None | None | None | None | None | None | None | None | None | None | None | None | None | None | None | None | None | None | 2353.65 | None | None | None | None | None | None | None | None | None | None | None | None |
| -1 | None | None | None | None | 3819.564 | None | None | None | None | None | None | None | None | 1016.77 | None | None | None | None | None | None | None | None | None | None | None | None | None | None | None | 4636.89 | None | None | None | None | None | None | 5788.29 | None | None | 1959.46 | None | None | None | None | None | None | None | None | None | None | None | None | None | None | None | None | None | None | None | None | 3991.64 | None | None | None | None | None | None | None | None | None | None | None | None | None | None | None | None | None | None | None | None | None | 2732.38 | None | None | None | None | None | None | None | None | None | None | 3688.37 | None |
| 0 | None | None | None | None | 4624.4 | None | None | 1955.54 | None | None | None | None | None | 1147.85 | None | None | None | None | 1726.59 | None | None | None | None | None | None | None | None | None | None | 4944.83 | None | None | None | None | None | None | 6133.29 | None | None | 2089.47 | None | None | None | None | None | None | None | None | None | None | None | None | None | None | None | None | None | None | None | None | 5557.23 | None | None | None | None | None | None | None | None | None | None | None | None | None | None | None | None | None | None | None | None | None | 3507.15 | None | None | None | None | None | None | None | None | None | None | 4555.4 | None |
| 1 | None | None | None | None | 5787.633 | None | None | 2140.58 | None | None | None | None | None | 1080.01 | None | None | None | None | 1995.0 | None | None | None | None | None | None | None | None | None | None | 5551.14 | None | None | None | None | None | None | 7130.33 | None | None | 2003.79 | None | None | None | None | None | None | None | None | None | None | None | None | None | None | None | None | None | None | None | None | 5818.07 | None | None | None | None | None | None | None | None | None | None | None | None | None | None | None | None | None | None | None | None | None | 3784.31 | None | None | None | None | None | None | None | None | None | None | 5624.99 | None |
| 2 | None | None | None | None | None | None | None | None | None | None | None | None | None | 714.45 | None | None | None | None | None | None | None | None | None | None | None | None | None | None | None | 6877.26 | None | None | None | None | None | None | 9407.11 | None | None | 2478.7 | None | None | None | None | None | None | None | None | None | None | None | None | None | None | None | None | None | None | None | None | 6873.34 | None | None | None | None | None | None | None | None | None | None | None | None | None | None | None | None | None | None | None | None | None | None | None | None | None | None | None | None | None | None | None | None | None | None |
| 3 | None | None | None | None | None | None | None | None | None | None | None | None | None | None | None | None | None | None | None | None | None | None | None | None | None | None | None | None | None | 8380.68 | None | None | None | None | None | None | None | None | None | 2489.44 | None | None | None | None | None | None | None | None | None | None | None | None | None | None | None | None | None | None | None | None | 8859.99 | None | None | None | None | None | None | None | None | None | None | None | None | None | None | None | None | None | None | None | None | None | None | None | None | None | None | None | None | None | None | None | None | None | None |
| 4 | None | None | None | None | None | None | None | None | None | None | None | None | None | None | None | None | None | None | None | None | None | None | None | None | None | None | None | None | None | 9748.43 | None | None | None | None | None | None | None | None | None | 2587.75 | None | None | None | None | None | None | None | None | None | None | None | None | None | None | None | None | None | None | None | None | None | None | None | None | None | None | None | None | None | None | None | None | None | None | None | None | None | None | None | None | None | None | None | None | None | None | None | None | None | None | None | None | None | None | None |
| 5 | None | None | None | None | None | None | None | None | None | None | None | None | None | None | None | None | None | None | None | None | None | None | None | None | None | None | None | None | None | 9904.6 | None | None | None | None | None | None | None | None | None | None | None | None | None | None | None | None | None | None | None | None | None | None | None | None | None | None | None | None | None | None | None | None | None | None | None | None | None | None | None | None | None | None | None | None | None | None | None | None | None | None | None | None | None | None | None | None | None | None | None | None | None | None | None | None | None |
| 6 | None | None | None | None | None | None | None | None | None | None | None | None | None | None | None | None | None | None | None | None | None | None | None | None | None | None | None | None | None | None | None | None | None | None | None | None | None | None | None | None | None | None | None | None | None | None | None | None | None | None | None | None | None | None | None | None | None | None | None | None | None | None | None | None | None | None | None | None | None | None | None | None | None | None | None | None | None | None | None | None | None | None | None | None | None | None | None | None | None | None | None | None | None | None | None |Time point (years)
Dialysis initiation
n
2
1
6
5
6
8
10
10
5
3
2
1
0

## Slide 5
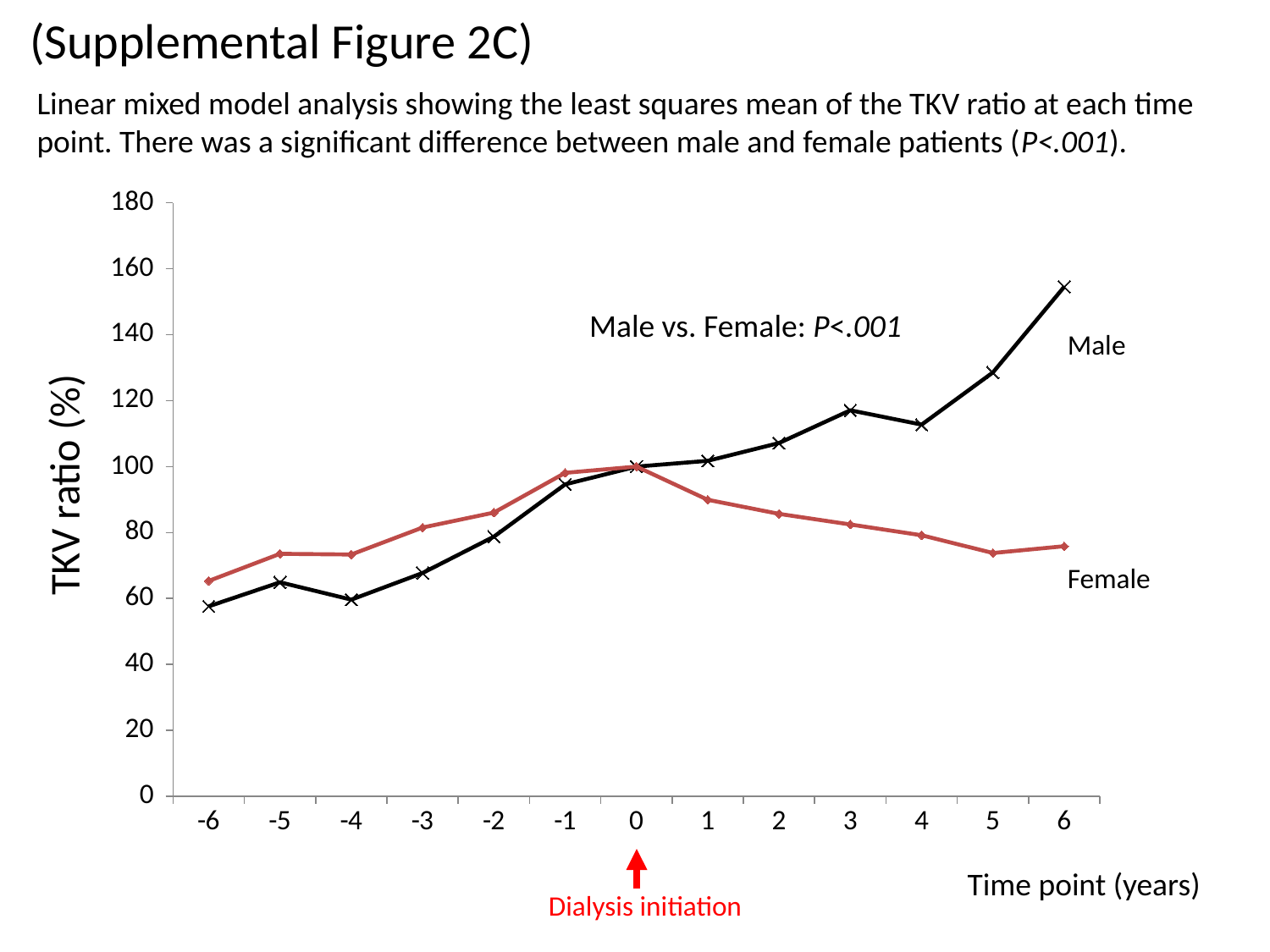

(Supplemental Figure 2C)
Linear mixed model analysis showing the least squares mean of the TKV ratio at each time point. There was a significant difference between male and female patients (P<.001).
### Chart
| Category | | |
|---|---|---|
| -6 | 57.606350385694256 | 65.30404082878505 |
| -5 | 64.89408946660677 | 73.54453239854134 |
| -4 | 59.64730109027812 | 73.33109204448448 |
| -3 | 67.71495894501294 | 81.52970323842504 |
| -2 | 78.74160262268806 | 86.05611184071992 |
| -1 | 94.61161351353307 | 98.0960636856664 |
| 0 | 100.0 | 100.0 |
| 1 | 101.75180236741838 | 89.93527157667496 |
| 2 | 107.142092606981 | 85.66553636918692 |
| 3 | 117.06391667853318 | 82.44987226700808 |
| 4 | 112.72187733182406 | 79.1647823732249 |
| 5 | 128.54795124226823 | 73.78634947705915 |
| 6 | 154.5210271056279 | 75.84566564439652 |Male vs. Female: P<.001
Male
TKV ratio (%)
Female
Time point (years)
Dialysis initiation

## Slide 6
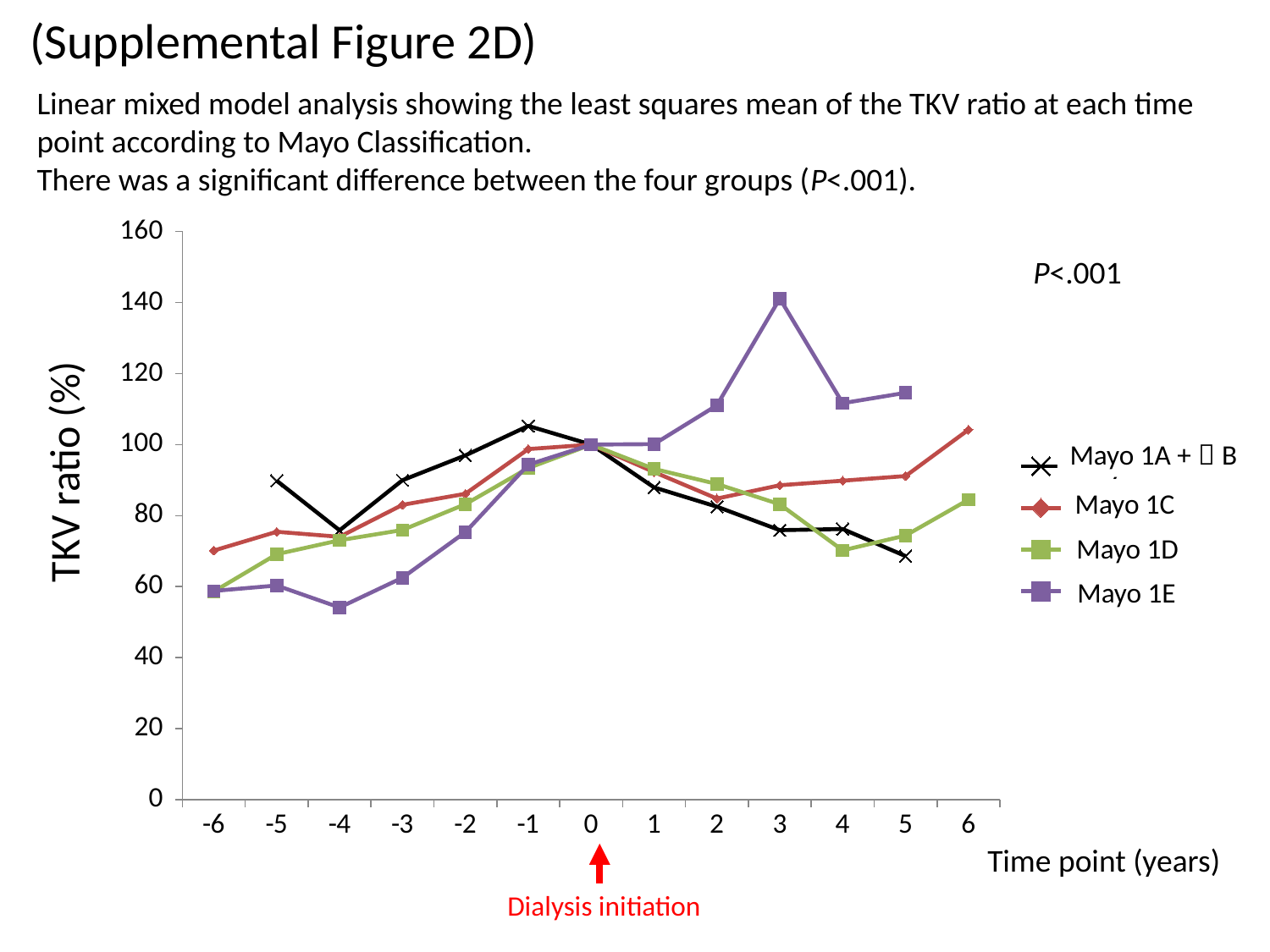

(Supplemental Figure 2D)
Linear mixed model analysis showing the least squares mean of the TKV ratio at each time point according to Mayo Classification.
There was a significant difference between the four groups (P<.001).
### Chart
| Category | | | | |
|---|---|---|---|---|
| -6 | None | 70.15918484368794 | 58.62168700259253 | 58.789522146253255 |
| -5 | 89.83650430648675 | 75.44122337017242 | 69.12787743082711 | 60.32675388593163 |
| -4 | 75.84388981007417 | 74.01906136416217 | 73.05323455702462 | 54.12617340437071 |
| -3 | 89.9677241727542 | 83.04060647049002 | 75.94658882684155 | 62.51201887084844 |
| -2 | 96.95110170462635 | 86.14256224236226 | 83.21517321456822 | 75.34709334339468 |
| -1 | 105.24920786547935 | 98.73674904550371 | 93.39471270075737 | 94.3445471111597 |
| 0 | 100.00000000000006 | 100.00000000000047 | 99.9999999999998 | 100.00000000000003 |
| 1 | 87.94338596176505 | 92.26686582357797 | 93.14589239273211 | 100.12441995397808 |
| 2 | 82.51860294014433 | 84.80192260515281 | 88.90034108692488 | 111.0442456376757 |
| 3 | 75.89217437756207 | 88.5555819058458 | 83.19747766461961 | 141.10881891239916 |
| 4 | 76.2158287715225 | 89.8385100747899 | 70.21559704766229 | 111.64465912690041 |
| 5 | 68.59422145777594 | 91.13764186414292 | 74.39017286853085 | 114.57256456885126 |
| 6 | None | 104.18366736661146 | 84.48357168071837 | None |P<.001
Mayo 1A +１B
TKV ratio (%)
Mayo 1C
Mayo 1D
Mayo 1E
Time point (years)
Dialysis initiation

## Slide 7
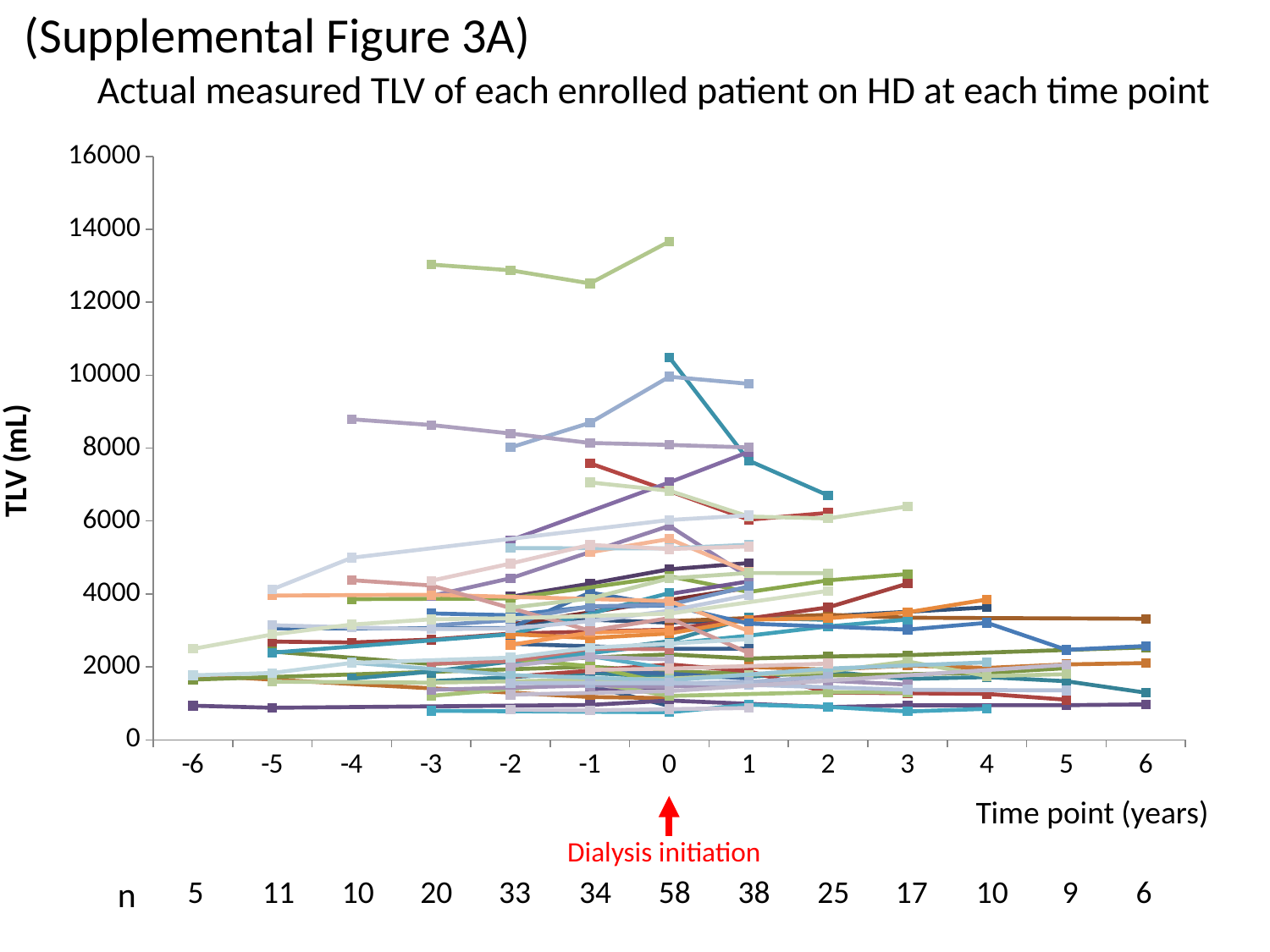

(Supplemental Figure 3A)
Actual measured TLV of each enrolled patient on HD at each time point
### Chart
| Category | | | | | | | | | | | | | | | | | | | | | | | | | | | | | | | | | | | | | | | | | | | | | | | | | | | | | | | | | | | | | | | | | | | | | | | | | | | | | | | | | | | | | | | | | | | | | | | |
|---|---|---|---|---|---|---|---|---|---|---|---|---|---|---|---|---|---|---|---|---|---|---|---|---|---|---|---|---|---|---|---|---|---|---|---|---|---|---|---|---|---|---|---|---|---|---|---|---|---|---|---|---|---|---|---|---|---|---|---|---|---|---|---|---|---|---|---|---|---|---|---|---|---|---|---|---|---|---|---|---|---|---|---|---|---|---|---|---|---|---|---|---|---|---|---|
| -6 | None | None | None | None | None | None | None | None | None | None | None | None | None | None | None | None | None | 1770.433 | None | None | 1652.17 | 936.175 | None | None | None | None | None | None | None | None | None | None | None | None | None | None | None | None | None | None | None | None | None | None | None | None | None | None | None | None | None | None | None | None | None | None | None | None | None | None | None | None | None | None | None | None | None | None | None | None | None | None | None | None | None | None | None | None | None | None | None | None | None | None | None | None | 2494.45 | None | 1770.23 | None | None | None | None | None | None |
| -5 | None | None | None | None | None | None | None | None | None | None | None | None | None | None | 2420.109 | None | None | None | None | None | None | 879.271 | None | None | None | 2693.495 | None | None | None | None | 3056.945 | None | None | None | 2390.938 | None | None | None | None | None | None | None | None | None | None | None | None | None | None | None | None | None | None | None | None | None | None | None | None | None | None | None | None | None | None | 3957.04 | None | None | 1596.18 | None | None | None | None | None | None | None | None | None | None | None | None | None | None | None | 3142.49 | None | 2888.79 | None | 1832.25 | None | 4126.88 | None | None | None | None |
| -4 | None | None | None | None | None | None | None | None | None | None | None | None | None | None | None | None | None | None | None | None | None | None | 1677.126 | None | None | 2669.456 | None | None | None | None | None | None | 3858.263 | None | None | None | None | None | None | None | None | None | None | None | None | None | None | None | None | None | None | None | None | None | None | None | None | None | None | None | None | None | None | None | None | None | None | 4376.69 | None | 8791.19 | 2114.94 | None | None | None | None | None | None | None | None | None | None | None | None | None | 3083.88 | None | 3159.75 | None | 2109.54 | None | 4992.28 | None | None | None | None |
| -3 | None | None | None | None | None | None | None | None | None | None | None | None | None | None | 2079.977 | None | 1605.5 | None | None | None | None | None | 1870.413 | None | None | 2748.1 | None | None | None | None | 3062.234 | None | None | None | None | None | None | None | None | None | None | None | 3465.99 | None | None | None | 796.8 | None | None | None | None | None | None | None | 3132.82 | 2089.16 | 1206.47 | 3945.11 | None | None | None | None | 13032.67 | 1372.48 | None | 3977.82 | None | 4231.29 | 1567.92 | 8632.67 | None | None | None | None | None | None | None | None | None | None | None | None | None | None | 3035.84 | None | 3304.27 | None | None | None | None | 4362.32 | None | None | None |
| -2 | 3183.618 | 3160.454 | None | 3924.266 | None | None | None | None | None | None | None | None | 2633.667 | None | None | None | None | None | None | None | None | None | 2148.06 | None | None | 2912.94 | None | None | None | None | 3065.87 | 1727.34 | 3875.06 | None | 2894.37 | None | None | None | None | None | 2115.0 | 2898.476 | 3416.08 | None | 2210.71 | None | None | None | None | None | None | 5471.56 | None | 2604.13 | 3281.92 | 2160.22 | None | 4430.81 | None | None | None | None | 12875.29 | None | None | None | 8017.3 | None | None | 8400.4 | 1776.45 | None | None | None | 3624.186 | 2025.417 | 5256.287 | None | 1550.294 | None | None | 1239.488 | None | None | 3052.39 | None | 3334.08 | 825.79 | 2255.37 | None | None | 4833.23 | None | None | None |
| -1 | 3293.096 | None | None | 4282.496 | None | None | 1479.344 | None | None | None | None | None | None | None | None | None | 1835.05 | 1173.91 | None | None | 2004.7 | 956.93 | None | None | 1905.73 | None | None | 1360.23 | None | None | 4038.96 | None | None | None | None | None | None | 7583.46 | None | None | None | 2791.381 | 3648.31 | None | 2020.06 | None | None | None | None | None | None | None | 2295.535 | 2939.56 | 3660.52 | 2497.21 | 1579.19 | None | None | None | None | None | 12516.29 | 1489.79 | None | None | 8693.33 | 2994.76 | None | 8140.93 | None | 5143.513 | 1547.705 | None | 3874.82 | 2278.269 | None | None | None | None | 7058.3 | None | None | None | 3252.25 | 1918.01 | None | 807.34 | 2524.07 | None | None | 5345.24 | None | None | None |
| 0 | 3214.725 | 3831.968 | None | 4676.339 | None | 3252.53 | 928.113 | None | None | None | None | None | 2496.37 | None | 2342.61 | None | 1716.61 | 1143.79 | None | None | 1871.63 | 1077.16 | 2703.96 | 1984.38 | 1812.85 | 3022.31 | None | 1361.9 | 10488.68 | None | 3666.57 | 2066.38 | 4483.98 | 3999.55 | 4024.9 | None | None | 6820.21 | None | None | 2634.514 | 2916.8 | 3683.56 | None | 1523.24 | None | 756.38 | None | None | None | None | 7058.71 | 1948.57 | 3002.52 | 3704.38 | 2487.91 | 1203.43 | 5866.02 | None | None | None | None | 13666.41 | 1465.27 | None | 3804.23 | 9958.43 | 3337.09 | 1677.72 | 8087.05 | 1629.8 | 5507.225 | 1545.599 | None | 4425.902 | 2204.286 | 5248.986 | None | 1568.947 | None | 6830.12 | 1335.945 | None | None | 3539.75 | 1953.22 | 3459.23 | 840.13 | 2638.57 | None | 6028.94 | 5230.45 | None | None | None |
| 1 | 3287.735 | 4170.673 | None | 4847.696 | None | None | None | None | None | None | None | None | 2500.48 | None | 2228.4 | None | 1728.75 | None | None | None | None | None | 3348.8 | 2002.61 | 1682.29 | None | None | None | 7657.49 | None | None | 1882.61 | 4053.42 | 4344.25 | None | None | None | 6036.7 | None | None | 2856.517 | 3295.89 | 3187.15 | None | None | None | 963.12 | None | None | None | None | 7897.98 | None | None | 4207.15 | None | None | 4466.75 | None | None | None | None | None | 1504.76 | None | 2986.96 | 9763.98 | 2382.17 | 1785.12 | 8017.73 | None | 4600.163 | 1585.625 | None | 4575.621 | None | 5349.583 | None | 1509.838 | None | 6123.05 | None | None | None | 3965.72 | None | None | 873.78 | 2760.23 | None | 6154.27 | 5301.07 | None | None | None |
| 2 | 3390.926 | None | None | None | None | 3429.35 | None | None | None | None | None | None | None | None | 2284.9 | None | 1869.45 | None | None | None | 1764.73 | 898.43 | 3289.83 | 1923.72 | None | 3631.79 | None | None | 6701.06 | None | None | 1296.95 | 4373.11 | None | None | None | None | 6223.03 | None | None | 3109.737 | 3334.59 | None | None | None | None | 903.01 | None | None | None | None | None | None | None | None | None | 1307.51 | None | None | None | None | None | None | 1620.31 | None | None | None | None | 1872.04 | None | 1953.62 | None | 1737.446 | None | 4569.12 | None | None | None | None | None | 6070.46 | None | None | None | None | 2090.2 | 4084.83 | None | None | None | None | None | None | None | None |
| 3 | None | None | None | None | None | 3347.92 | None | None | None | None | None | None | None | None | 2321.41 | None | 1670.74 | None | None | None | None | 946.71 | None | 2035.72 | None | 4283.98 | None | None | None | None | None | 1277.94 | 4546.08 | None | None | None | None | None | None | None | 3298.85 | 3498.05 | 3021.41 | None | None | None | 777.53 | None | None | None | None | None | None | None | None | None | 1299.14 | None | None | None | None | None | None | 1515.32 | None | None | None | None | 2148.93 | None | None | None | None | None | None | None | None | None | 1372.75 | None | 6401.55 | None | None | None | None | None | None | None | None | None | None | None | None | None | None |
| 4 | 3631.98 | None | None | None | None | None | None | None | None | None | None | None | None | None | None | None | 1719.95 | None | None | None | 1836.93 | None | None | 1970.54 | None | None | None | None | None | None | None | 1259.87 | None | None | None | None | None | None | None | None | None | 3848.1 | 3208.73 | None | None | None | 849.77 | None | None | None | None | None | None | None | None | None | None | None | None | None | None | None | None | None | None | None | None | None | 1748.17 | None | 2124.95 | None | None | None | None | None | None | None | None | None | None | None | None | None | None | None | None | None | None | None | None | None | None | None | None |
| 5 | None | None | None | None | None | None | None | None | None | None | None | None | None | None | None | None | 1606.94 | None | None | None | 1964.91 | 950.7 | None | 2068.71 | None | None | None | None | None | None | None | 1099.42 | None | None | None | None | None | None | None | None | None | None | 2467.97 | None | None | None | None | None | None | None | None | None | None | None | None | None | None | None | None | None | None | None | None | None | None | None | None | None | 1800.4 | None | None | None | None | None | None | None | None | None | 1356.44 | None | None | 2057.91 | None | None | None | None | None | None | None | None | None | None | None | None | None |
| 6 | None | None | None | None | None | 3319.47 | None | None | None | None | None | None | None | None | 2536.05 | None | 1292.8 | None | None | None | None | 971.58 | None | 2104.63 | None | None | None | None | None | None | None | None | None | None | None | None | None | None | None | None | None | None | 2572.81 | None | None | None | None | None | None | None | None | None | None | None | None | None | None | None | None | None | None | None | None | None | None | None | None | None | None | None | None | None | None | None | None | None | None | None | None | None | None | None | None | None | None | None | None | None | None | None | None | None | None | None | None |Time point (years)
Dialysis initiation
n
5
11
10
20
33
34
58
38
25
17
10
9
6

## Slide 8
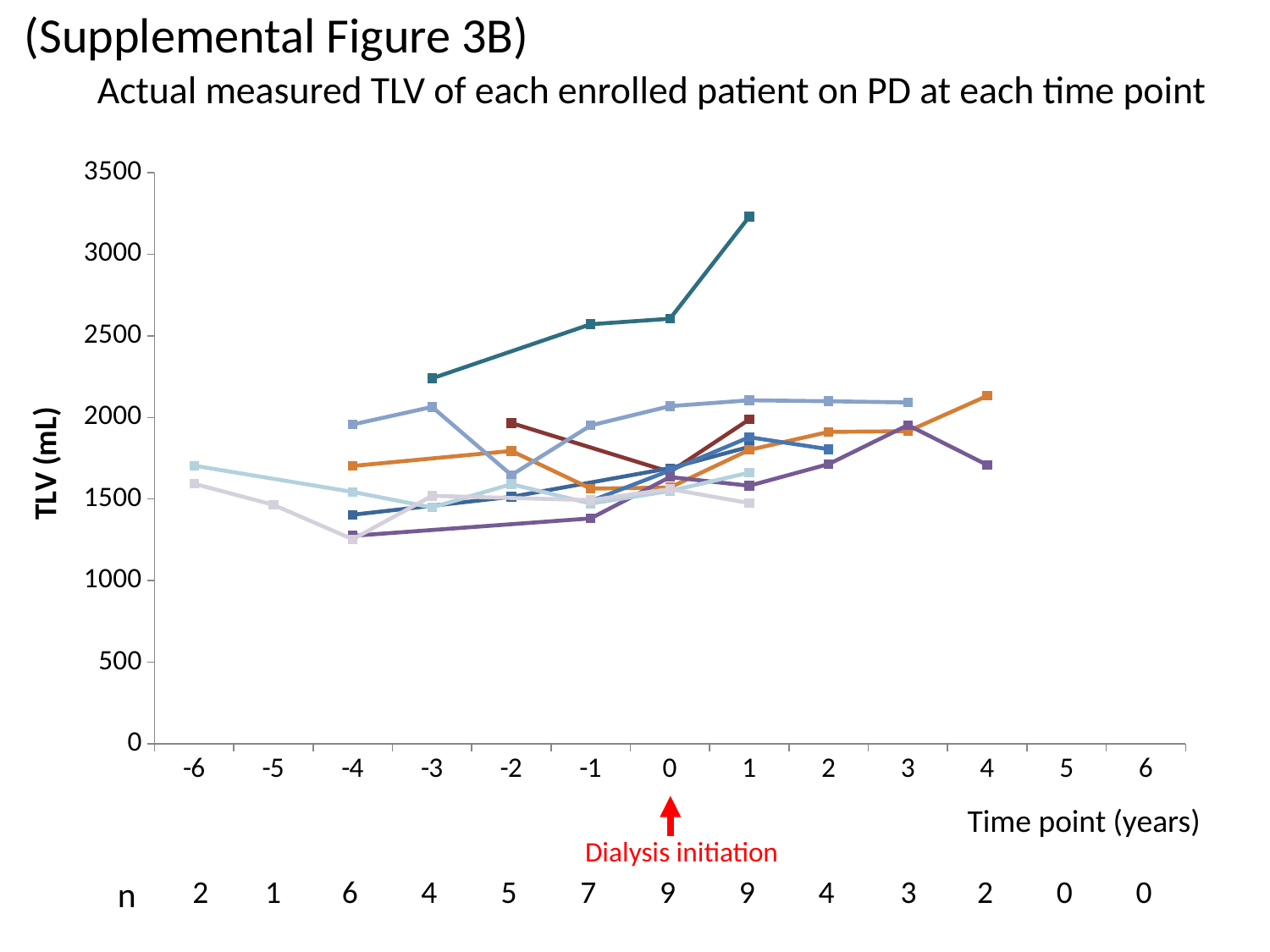

(Supplemental Figure 3B)
Actual measured TLV of each enrolled patient on PD at each time point
### Chart
| Category | | | | | | | | | | | | | | | | | | | | | | | | | | | | | | | | | | | | | | | | | | | | | | | | | | | | | | | | | | | | | | | | | | | | | | | | | | | | | | | | | | | | | | | | | | | | | | | |
|---|---|---|---|---|---|---|---|---|---|---|---|---|---|---|---|---|---|---|---|---|---|---|---|---|---|---|---|---|---|---|---|---|---|---|---|---|---|---|---|---|---|---|---|---|---|---|---|---|---|---|---|---|---|---|---|---|---|---|---|---|---|---|---|---|---|---|---|---|---|---|---|---|---|---|---|---|---|---|---|---|---|---|---|---|---|---|---|---|---|---|---|---|---|---|---|
| -6 | None | None | None | None | None | None | None | None | None | None | None | None | None | None | None | None | None | None | None | None | None | None | None | None | None | None | None | None | None | None | None | None | None | None | None | None | None | None | None | None | None | None | None | None | None | None | None | None | None | None | None | None | None | None | None | None | None | None | None | None | None | None | None | None | None | None | None | None | None | None | None | None | None | None | None | None | None | None | None | None | None | None | 1704.69 | None | None | None | None | None | None | None | None | None | None | 1593.32 | None |
| -5 | None | None | None | None | None | None | None | None | None | None | None | None | None | None | None | None | None | None | None | None | None | None | None | None | None | None | None | None | None | None | None | None | None | None | None | None | None | None | None | None | None | None | None | None | None | None | None | None | None | None | None | None | None | None | None | None | None | None | None | None | None | None | None | None | None | None | None | None | None | None | None | None | None | None | None | None | None | None | None | None | None | None | None | None | None | None | None | None | None | None | None | None | None | 1464.38 | None |
| -4 | None | None | None | None | None | None | None | None | None | None | None | None | None | None | None | None | None | None | 1403.768 | None | None | None | None | None | None | None | None | None | None | 1702.69 | None | None | None | None | None | None | None | None | None | 1274.453 | None | None | None | None | None | None | None | None | None | None | None | None | None | None | None | None | None | None | None | None | 1956.68 | None | None | None | None | None | None | None | None | None | None | None | None | None | None | None | None | None | None | None | None | None | 1543.13 | None | None | None | None | None | None | None | None | None | None | 1253.41 | None |
| -3 | None | None | None | None | 2239.224 | None | None | None | None | None | None | None | None | None | None | None | None | None | None | None | None | None | None | None | None | None | None | None | None | None | None | None | None | None | None | None | None | None | None | None | None | None | None | None | None | None | None | None | None | None | None | None | None | None | None | None | None | None | None | None | 2064.34 | None | None | None | None | None | None | None | None | None | None | None | None | None | None | None | None | None | None | None | None | None | 1448.32 | None | None | None | None | None | None | None | None | None | None | 1519.64 | None |
| -2 | None | None | None | None | None | None | None | 1965.565 | None | None | None | None | None | None | None | None | None | None | 1513.33 | None | None | None | None | None | None | None | None | None | None | 1794.95 | None | None | None | None | None | None | None | None | None | None | None | None | None | None | None | None | None | None | None | None | None | None | None | None | None | None | None | None | None | None | 1646.74 | None | None | None | None | None | None | None | None | None | None | None | None | None | None | None | None | None | None | None | None | None | 1590.89 | None | None | None | None | None | None | None | None | None | None | None | None |
| -1 | None | None | None | None | 2570.787 | None | None | None | None | None | None | None | None | None | None | None | None | None | None | None | None | None | None | None | None | None | None | None | None | 1563.41 | None | None | None | None | None | None | 1487.59 | None | None | 1381.4 | None | None | None | None | None | None | None | None | None | None | None | None | None | None | None | None | None | None | None | None | 1950.87 | None | None | None | None | None | None | None | None | None | None | None | None | None | None | None | None | None | None | None | None | None | 1472.02 | None | None | None | None | None | None | None | None | None | None | 1493.08 | None |
| 0 | None | None | None | None | 2604.553 | None | None | 1665.63 | None | None | None | None | None | None | None | None | None | None | 1689.31 | None | None | None | None | None | None | None | None | None | None | 1570.53 | None | None | None | None | None | None | 1674.72 | None | None | 1634.37 | None | None | None | None | None | None | None | None | None | None | None | None | None | None | None | None | None | None | None | None | 2069.03 | None | None | None | None | None | None | None | None | None | None | None | None | None | None | None | None | None | None | None | None | None | 1549.04 | None | None | None | None | None | None | None | None | None | None | 1560.66 | None |
| 1 | None | None | None | None | 3229.998 | None | None | 1987.96 | None | None | None | None | None | None | None | None | None | None | 1820.0 | None | None | None | None | None | None | None | None | None | None | 1800.94 | None | None | None | None | None | None | 1878.75 | None | None | 1581.16 | None | None | None | None | None | None | None | None | None | None | None | None | None | None | None | None | None | None | None | None | 2104.85 | None | None | None | None | None | None | None | None | None | None | None | None | None | None | None | None | None | None | None | None | None | 1662.06 | None | None | None | None | None | None | None | None | None | None | 1474.95 | None |
| 2 | None | None | None | None | None | None | None | None | None | None | None | None | None | None | None | None | None | None | None | None | None | None | None | None | None | None | None | None | None | 1911.51 | None | None | None | None | None | None | 1805.12 | None | None | 1713.96 | None | None | None | None | None | None | None | None | None | None | None | None | None | None | None | None | None | None | None | None | 2099.16 | None | None | None | None | None | None | None | None | None | None | None | None | None | None | None | None | None | None | None | None | None | None | None | None | None | None | None | None | None | None | None | None | None | None |
| 3 | None | None | None | None | None | None | None | None | None | None | None | None | None | None | None | None | None | None | None | None | None | None | None | None | None | None | None | None | None | 1915.37 | None | None | None | None | None | None | None | None | None | 1953.15 | None | None | None | None | None | None | None | None | None | None | None | None | None | None | None | None | None | None | None | None | 2091.68 | None | None | None | None | None | None | None | None | None | None | None | None | None | None | None | None | None | None | None | None | None | None | None | None | None | None | None | None | None | None | None | None | None | None |
| 4 | None | None | None | None | None | None | None | None | None | None | None | None | None | None | None | None | None | None | None | None | None | None | None | None | None | None | None | None | None | 2132.07 | None | None | None | None | None | None | None | None | None | 1708.39 | None | None | None | None | None | None | None | None | None | None | None | None | None | None | None | None | None | None | None | None | None | None | None | None | None | None | None | None | None | None | None | None | None | None | None | None | None | None | None | None | None | None | None | None | None | None | None | None | None | None | None | None | None | None | None |
| 5 | None | None | None | None | None | None | None | None | None | None | None | None | None | None | None | None | None | None | None | None | None | None | None | None | None | None | None | None | None | None | None | None | None | None | None | None | None | None | None | None | None | None | None | None | None | None | None | None | None | None | None | None | None | None | None | None | None | None | None | None | None | None | None | None | None | None | None | None | None | None | None | None | None | None | None | None | None | None | None | None | None | None | None | None | None | None | None | None | None | None | None | None | None | None | None |
| 6 | None | None | None | None | None | None | None | None | None | None | None | None | None | None | None | None | None | None | None | None | None | None | None | None | None | None | None | None | None | None | None | None | None | None | None | None | None | None | None | None | None | None | None | None | None | None | None | None | None | None | None | None | None | None | None | None | None | None | None | None | None | None | None | None | None | None | None | None | None | None | None | None | None | None | None | None | None | None | None | None | None | None | None | None | None | None | None | None | None | None | None | None | None | None | None |Time point (years)
Dialysis initiation
n
2
1
6
4
5
7
9
9
4
3
2
0
0

## Slide 9
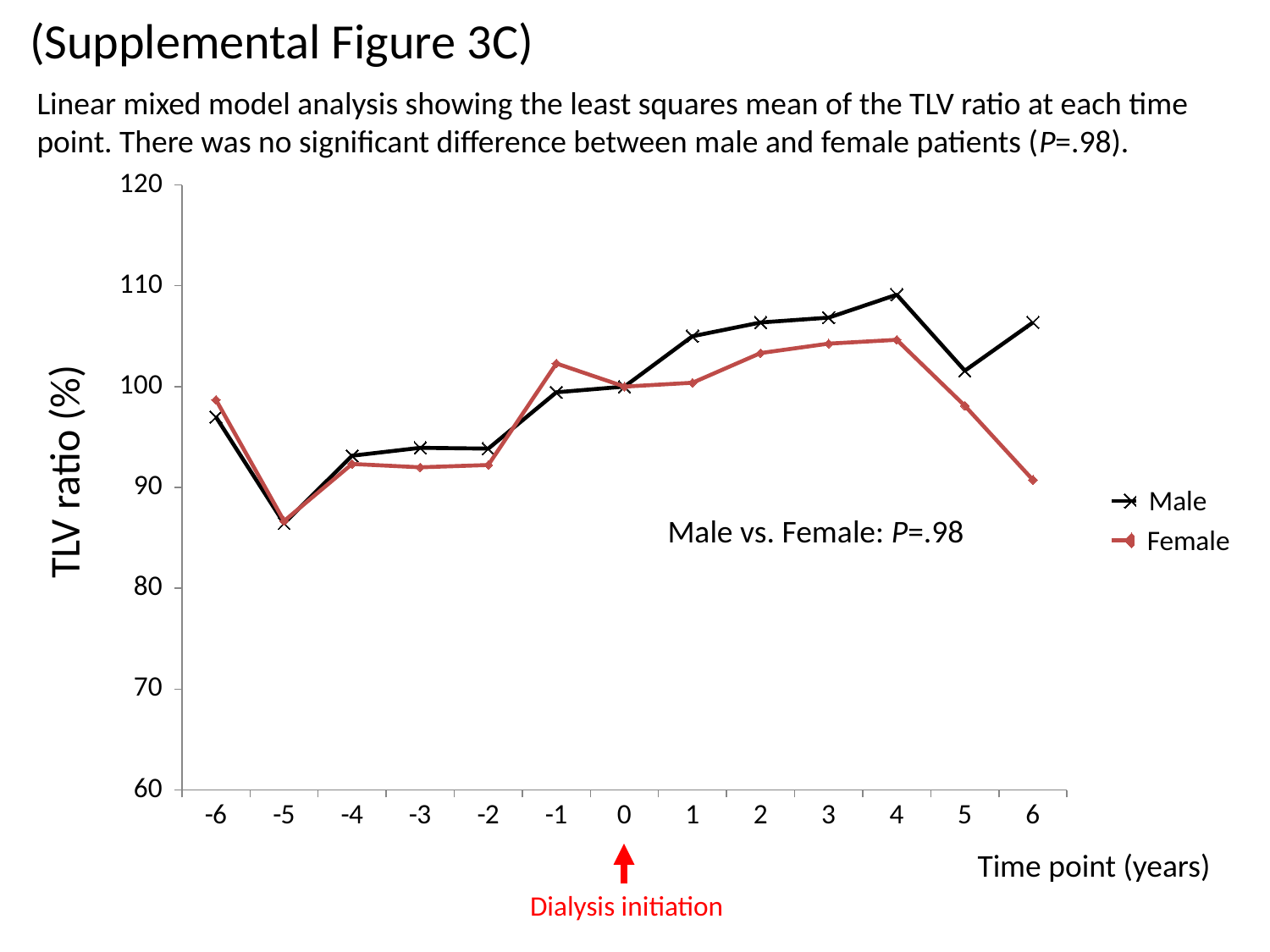

(Supplemental Figure 3C)
Linear mixed model analysis showing the least squares mean of the TLV ratio at each time point. There was no significant difference between male and female patients (P=.98).
### Chart
| Category | | |
|---|---|---|
| -6 | 96.98837454281617 | 98.6932925988319 |
| -5 | 86.4262695696556 | 86.66599013432517 |
| -4 | 93.14407294455543 | 92.3282682752313 |
| -3 | 93.93112328702047 | 91.99516883884542 |
| -2 | 93.84925424201938 | 92.22260537827138 |
| -1 | 99.43278577342448 | 102.29522776929248 |
| 0 | 99.99999999999983 | 100.00000000000003 |
| 1 | 104.99563862628632 | 100.37854898893326 |
| 2 | 106.3572417184534 | 103.32568095387074 |
| 3 | 106.83916978767991 | 104.25915848501387 |
| 4 | 109.1121853056048 | 104.63764219023194 |
| 5 | 101.57471413651004 | 98.09879439923733 |
| 6 | 106.36119368106384 | 90.74344413154077 |TLV ratio (%)
Male
Male vs. Female: P=.98
Female
Time point (years)
Dialysis initiation

## Slide 10
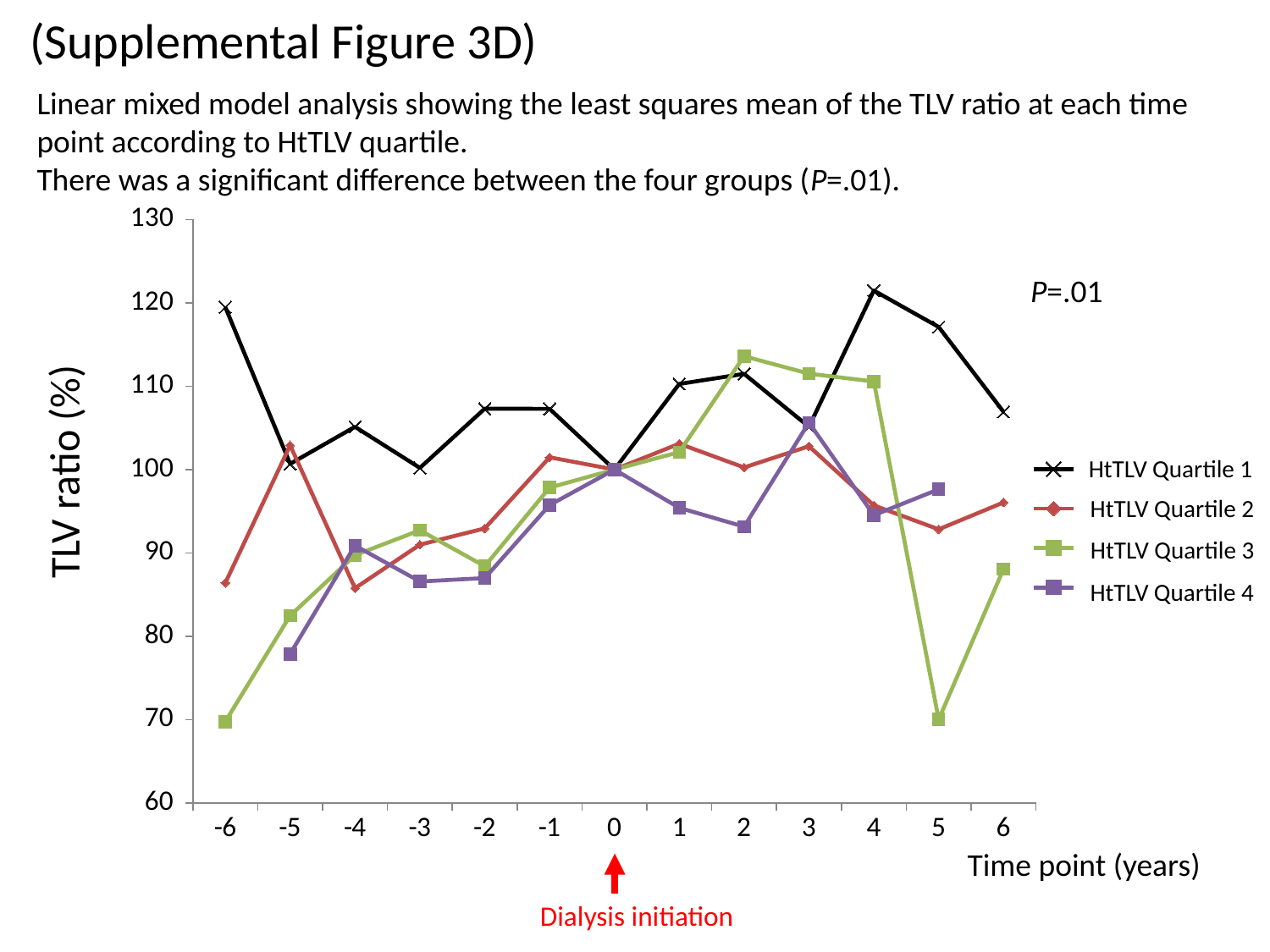

(Supplemental Figure 3D)
Linear mixed model analysis showing the least squares mean of the TLV ratio at each time point according to HtTLV quartile.
There was a significant difference between the four groups (P=.01).
### Chart
| Category | | | | |
|---|---|---|---|---|
| -6 | 119.48376526659317 | 86.38721356085786 | 69.75208056300707 | None |
| -5 | 100.68604851563536 | 102.91199653106987 | 82.46681067901814 | 77.88005134394933 |
| -4 | 105.12869994528891 | 85.75865766734445 | 89.72411522234995 | 90.87270087678209 |
| -3 | 100.1996253606713 | 90.98077589082109 | 92.70929848771522 | 86.56328521315416 |
| -2 | 107.30139657580798 | 92.94015650396659 | 88.40051235860325 | 86.97110739995009 |
| -1 | 107.2880745344648 | 101.46989371019065 | 97.83511847548854 | 95.7369792329666 |
| 0 | 100.0 | 100.0 | 100.0 | 100.00000000000009 |
| 1 | 110.27182586605474 | 103.09823383469129 | 102.09264684558926 | 95.40969545175358 |
| 2 | 111.48269494103786 | 100.24928830080417 | 113.59916170934824 | 93.1472551294403 |
| 3 | 105.12795242787907 | 102.80208935247568 | 111.50744278339245 | 105.63842585530554 |
| 4 | 121.4800148216338 | 95.69662474301667 | 110.56358154468165 | 94.51856313700227 |
| 5 | 117.10725694611753 | 92.8235601368702 | 70.01094111092583 | 97.6317167025547 |
| 6 | 106.9206451967166 | 96.05717001828818 | 88.06914016542196 | None |P=.01
TLV ratio (%)
HtTLV Quartile 1
HtTLV Quartile 2
HtTLV Quartile 3
HtTLV Quartile 4
Time point (years)
Dialysis initiation

## Slide 11
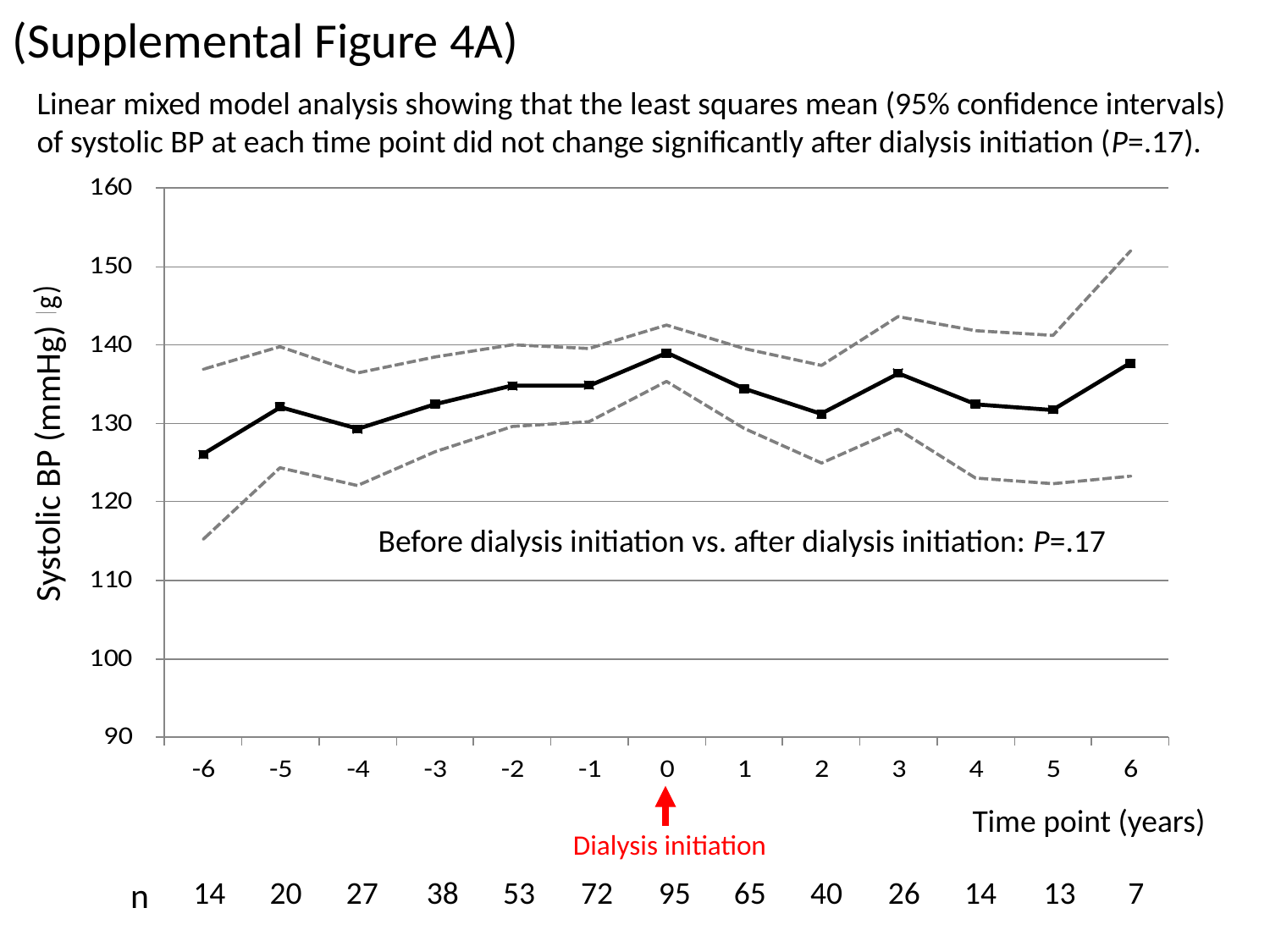

(Supplemental Figure 4A)
Linear mixed model analysis showing that the least squares mean (95% confidence intervals) of systolic BP at each time point did not change significantly after dialysis initiation (P=.17).
Systolic BP (mmHg)
Before dialysis initiation vs. after dialysis initiation: P=.17
Time point (years)
Dialysis initiation
n
14
20
27
38
53
72
95
65
40
26
14
13
7

## Slide 12
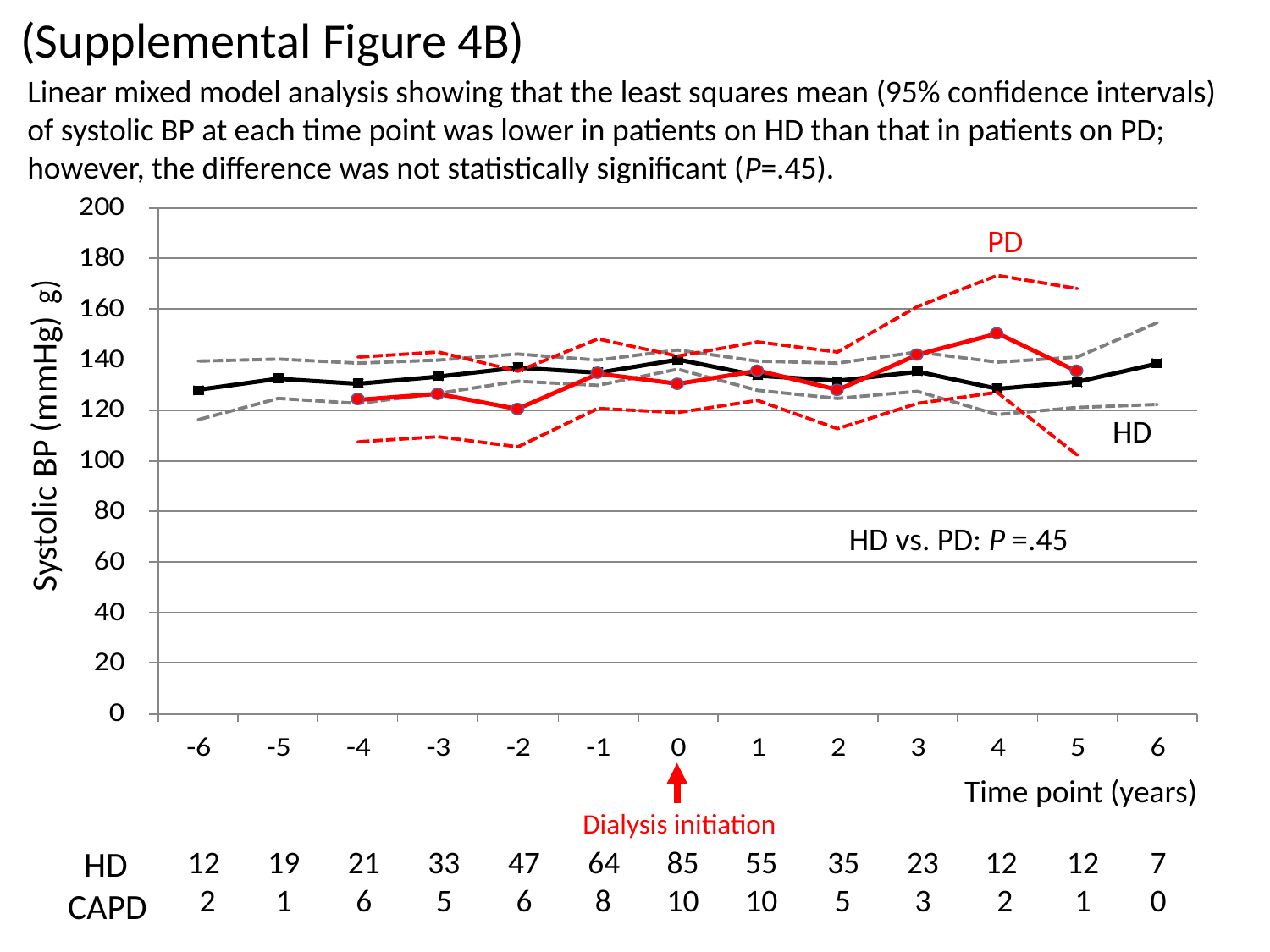

(Supplemental Figure 4B)
Linear mixed model analysis showing that the least squares mean (95% confidence intervals) of systolic BP at each time point was lower in patients on HD than that in patients on PD; however, the difference was not statistically significant (P=.45).
PD
HD
Systolic BP (mmHg)
HD vs. PD: P =.45
Time point (years)
Dialysis initiation
 HD
CAPD
12
 2
19
1
21
6
33
5
47
6
64
 8
85
10
55
10
35
 5
23
3
12
 2
12
1
7
0
